# Gut-liver axis in liver pre-metastatic niche formation: immune networks, microbial, and metabolic and emerging therapeutic opportunities

**DOI:** 10.3389/fmicb.2026.1931054

**Published:** 2026-10-15

**Authors:** Leyi Zhao, Qiutong Lin, Danyuan Zhang, Yani Liu, Mingtong Zong, Lingze Xi, Feiyu Xie, Shuyi Song, Peng Xue, Shijie Zhu, Xuelei Chu

**Affiliations:** 1 Department of Oncology, Wangjing Hospital of China Academy of Traditional Chinese Medicine, Beijing, China; 2 Graduate School, Beijing University of Chinese Medicine, Beijing, China; 3 Qi - Huang Chinese Medicine School, Beijing University of Chinese Medicine, Beijing, China; 4 Department of Integrated Traditional Chinese and Western Medicine Oncology, China-Japan Friendship Hospital, Beijing, China; 5 The Cancer Hospital of the University of Chinese Academy of Sciences (Zhejiang Cancer Hospital), Zhejiang, China; 6 Affiliated Hospital of Qingdao University, Qingdao, Shandong, China; 7 Department of Oncology, Guang’anmen Hospital, China Academy of Chinese Medical Sciences, Beijing, China

**Keywords:** cellular regulatory networks, gut-liver axis, intestinal microbiota, liver metastasis, metabolites, pre-metastatic niche

## Abstract

The liver is a leading target for distant metastasis across multiple cancer types, and liver metastasis invariably portends poor prognosis. The formation of pre-metastatic niches (PMNs) within the hepatic microenvironment creates a permissive “soil” for disseminated tumor cells. However, how the gut-liver axis integrates microbial and metabolic signals to remodel the hepatic PMN remains incompletely understood. In this review, we delineate the six hallmark features of PMN assembly, with an emphasis on the complex crosstalk among gut microbiota, their bioactive metabolites, and resident liver cells—including hepatocytes and hepatic immune populations. Notably, these elements engage in a dynamic, reciprocally regulated network characterized by bidirectional feedback loops, rather than a linear signaling cascade. We further survey the emerging landscape of diagnostic biomarkers, including exosomal microRNAs and neutrophil extracellular traps, alongside microbiota-targeted therapeutic interventions. This framework provides testable hypotheses for early interception of liver metastasis and highlights precision medicine opportunities targeting the gut-liver-immune axis.

## Graphical abstract

**Figure:**
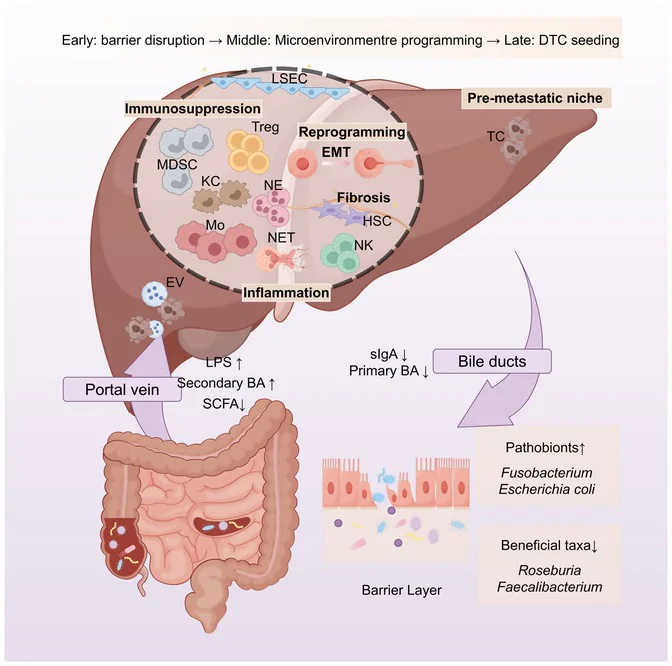

## 1 Background

The 2024 report from the World Health Organization’s International Agency for Research on Cancer estimates 20 million new cancer cases and 9.7 million cancer-related deaths globally (World Health Organization, 2024).

Metastasis, a hallmark of malignancy, remains the primary cause of cancer mortality. Among distant metastatic sites, the liver is a predominant target with dismal prognosis across multiple cancer types, and hepatic metastasis consistently confers a poor prognosis. Liver metastasis is also frequent in colorectal cancer (CRC), gastric, pancreatic, and lung cancer and in advanced breast cancer, with median survival typically measured in months (Engstrand et al., 2018; Qiu et al., 2018; Riihimäki et al., 2014; DeWitt et al., 2010; Wang et al., 2019). These figures underscore that liver metastasis remains a clinical challenge in which prevention-rather than treatment of established lesions-is an unmet need.

In 1889, Stephen Paget proposed the “seed and soil” theory, positing that metastasis depends on compatible interactions between cancer cell “seeds” and organ-specific microenvironmental “soil” (Kaplan et al., 2005). This concept was later extended by the discovery of the pre-metastatic niche (PMN)—a specialized microenvironment established in distant organs before overt metastasis (Liu and Cao, 2016). PMN formation involves primary tumor-derived factors such as TGF-β and VEGF, recruitment of bone marrow-derived cells, and remodeling of local stroma, collectively creating a permissive “soil” for disseminated tumor cells (DTCs). Hepatic PMN formation involves primary tumor-derived soluble factors and extracellular vesicles (EVs), recruitment of bone marrow-derived cells, and remodeling of local stroma and vasculature. Its cardinal features include inflammation, vascular remodeling, immunosuppression, and metabolic reprogramming, which together render the liver receptive to disseminated tumor cells (Liu and Cao, 2016).

The liver is anatomically positioned to receive intestinal signals: approximately 75% of its blood supply drains from the portal vein, conveying gut-derived nutrients, microbial products, and metabolites to hepatic cells (Wei et al., 2025). This anatomical arrangement defines the gut–liver axis, through which bile acids, short-chain fatty acids, lipopolysaccharide (LPS), and other microbial metabolites continuously modulate hepatic immunity and metabolism. In tumor-bearing hosts, primary tumor-associated gut dysbiosis disrupts this homeostatic exchange, and portal venous signals shift from tolerogenic to pro-inflammatory. These signals act on hepatocytes, liver sinusoidal endothelial cells, Kupffer cells (KCs), and recruited immune populations, reprogramming them toward a pro-metastatic phenotype and thereby initiating hepatic PMN formation.

There is growing evidence that the gut-liver axis plays a pivotal role in reprogramming the liver microenvironment to facilitate metastasis. Intestinal microbiota and their metabolites can travel via the portal vein to the liver, where they modulate hepatocytes, sinusoidal endothelial cells, KCs, and various immune cell populations, thereby establishing a pro-metastatic PMN.

Although the gut microbiota is increasingly recognized as a modifier of cancer progression, existing reviews have tended to treat microbial dysbiosis and liver metastasis as separate topics, and few integrate microbial ecology, metabolite signaling, and the hepatic cellular response into a mechanistic framework that explains how the gut–liver axis builds the hepatic PMN.

In this review, we synthesize evidence on how primary tumor-associated gut dysbiosis drives hepatic PMN formation. We first describe microbial dysbiosis and its metabolomic consequences as the upstream signal layer. We then dissect the cellular networks that sense these signals and execute PMN formation. We next organize microbiota-derived signaling pathways. Finally, we discuss emerging diagnostic biomarkers and microbiota-targeted interventions.

## 2 The gut-liver axis as the upstream driver: microbial dysbiosis

### 2.1 Gut microbiota dysbiosis in primary tumors

A growing body of clinical metagenomic and metabolomic evidence establishes that primary tumors across multiple anatomical sites are accompanied by characteristic gut microbiota dysbiosis and concomitant metabolomic remodeling, with changes that often emerge early in tumorigenesis and intensify with disease progression.

In CRC-the most extensively studied malignancy in this context-Yachida et al. (2019) conducted a landmark multi-omics analysis of 616 individuals undergoing colonoscopy, integrating fecal metagenomic sequencing with metabolomic profiling. They demonstrated that microbial and metabolomic shifts are detectable even at the stage of multiple polypoid adenomas and intramucosal carcinomas. Two distinct patterns of microbial enrichment emerged: *Fusobacterium nucleatum* (Fn) species were continuously and significantly elevated from intramucosal carcinoma through more advanced stages, whereas *Atopobium parvulum* and *Actinomyces odontolyticus*-which co-occurred in intramucosal carcinomas—were increased specifically in adenomatous and early cancerous lesions. Metabolomically, branched-chain amino acids and phenylalanine were significantly elevated in intramucosal carcinoma, while bile acids including deoxycholate accumulated in adenomas and early carcinomas, indicating that dysbiosis and metabolic reprogramming precede invasive disease (Yachida et al., 2019). Complementing this single-cohort finding, Thomas et al. (2019) performed a cross-cohort metagenomic analysis of multiple CRC datasets and identified reproducible microbial diagnostic signatures, notably a link between CRC and enrichment of microbial choline degradation pathways, alongside depletion of butyrate-producing taxa. A parallel meta-analysis by Wirbel et al. (2019) of eight geographically distinct cohorts further defined global CRC-specific microbial signatures, including consistent enrichment of Fn and *Porphyromonas asaccharolytica* and reduction of butyrate producers such as *Roseburia* and *Eubacterium* Critically, these dysbiotic profiles differ between non-metastatic and metastatic disease: Deng et al. (2025) compared 16 non-metastatic and 16 distant metastatic CRC patients using 16S rRNA sequencing and liquid chromatography-mass spectrometry-based untargeted metabolomics, revealing that metastatic patients harbored enrichment of *Pyramidobacter*, *Christensenellaceae_R-7_group*, and *Romboutsia*, while non-metastatic patients showed higher relative abundances of *Butyricicoccus*, *Ruminococcus_1*, and *Coprococcus_2*. A total of 91 differential metabolites were identified, predominantly involving nucleic acid, alkaloid, and lipid metabolism, with pathway enrichment in aminoacyl-tRNA biosynthesis, central carbon metabolism in cancer, phenylalanine metabolism, and vitamin B6 metabolism (Deng et al., 2025).

This dysbiosis-metabolome axis extends well beyond CRC. In breast cancer, Li et al. (2026) employed shotgun metagenomic sequencing of fecal samples from 38 stage I-III patients and 36 age- and BMI-matched healthy controls, revealing depletion of beneficial taxa including *Limosilactobacillus fermentum* and *Blautia* species, enrichment of *Prevotella copri*, and reduced short-chain fatty acid (butyrate, propionate) and purine metabolism pathways. Predictive metabolic profiling further indicated depletion of butyrate, propionate, purine intermediates (hypoxanthine, xanthine), and nicotinate in patients, and a machine learning model integrating microbial and metabolic features achieved an area under the curve of 0.78 in the discovery cohort and 0.73 in an independent validation cohort (Li et al., 2026). Earlier work by Goedert et al. (2015) had similarly reported reduced gut microbial diversity and increased Clostridiales in breast cancer patients relative to controls, with alterations in estrogen-metabolizing bacterial communities.

In non-small cell lung cancer, Liu et al. profiled 52 patients across TNM stages and 29 healthy controls using 16S rRNA gene sequencing combined with untargeted gas chromatography- and liquid chromatography-mass spectrometry metabolomics. While alpha-diversity did not differ consistently across stages, compositional shifts were pronounced: *Lachnospira* and *Blautia* were more abundant in healthy controls; *Bacteroides* and *Bacteroidaceae* predominated in stage T2; Prevotella dominated in stage T4; and *Ruminococcus gnavus* was notably elevated in stages N3 and M. Metabolomically, healthy controls showed higher levels of quinic acid and 3-hydroxybenzoic acid; stage I + II patients exhibited reduced L-cystathionine; and stage III + IV patients had increased quinic acid, phthalic acid, and L-lactic acid. Differentially abundant metabolites were enriched in β-alanine metabolism, phenylalanine/tyrosine/tryptophan biosynthesis, vitamin digestion and absorption, and glycine/serine/threonine metabolism (Liu et al., 2025).

Gastric cancer likewise displays progressive gut microbial dysbiosis. Jeong et al. (2024) used whole-metagenome shotgun sequencing to identify stage-specific microbiome signatures, finding that pathogenic Fusobacteria and species *Bacteroides caccae* and *Streptococcus anginosus* increased with disease progression, while *Bifidobacterium longum* and *Lachnospiraceae* bacteria were depleted. Nath and Natarajan (2024) independently identified *Akkermansia*, Gammaproteobacteria, and *Veillonella* as enriched taxa in gastric cancer patients with potential as non-invasive biomarkers. In pancreatic ductal adenocarcinoma (PDAC), Kartal et al. (2022) defined a fecal microbiota signature with high specificity for pancreatic cancer across multi-center cohorts, capable of distinguishing PDAC from healthy controls and other gastrointestinal malignancies. Mechanistically, Tintelnot et al. (2023) demonstrated that the microbiota-derived tryptophan metabolite indole-3-acetic acid (3-IAA) is clinically relevant in PDAC: high 3-IAA levels were associated with improved chemotherapy response, and 3-IAA enhanced treatment efficacy through neutrophil myeloperoxidase-dependent reactive oxygen species metabolism, directly linking a gut microbial metabolite to therapeutic outcome.

Across these cancer types, convergent themes emerge: depletion of short-chain fatty acid–producing and anti-inflammatory taxa, enrichment of pro-inflammatory and pathobiont species, and consistent alterations in bile acid, tryptophan, amino acid, and lipid metabolism.

### 2.2 The contribution of gut microbiota dysbiosis to the liver pre-metastatic niche

The liver and intestine maintain a dynamic bidirectional communication network, with 75% of hepatic blood supply derived from portal venous return transporting intestinal contents (Tripathi et al., 2018). Intestinal microbiota and their metabolites—including short-chain fatty acids, bile acids, and lipopolysaccharides—regulate systemic immunity and metabolic homeostasis, shuttling between the intestine and liver via the enterohepatic circulation (Zhang et al., 2024). Under physiological conditions, these signals sustain hepatic immune tolerance and metabolic homeostasis. In tumor-bearing hosts, however, the gut microbiota undergoes profound compositional and metabolic shifts, and the signals conveyed via portal venous blood transition from a physiological to a pathological state, thereby serving as the primary instigator of hepatic PMN formation.

In tumor-bearing hosts, the primary colorectal tumor drives gut microbiota dysbiosis, characterized by a markedly increased abundance of *Proteus mirabilis*, a markedly decreased abundance of *Bacteroides vulgatus*, and enrichment of opportunistic pathogens such as Fn and tumor-resident *Escherichia coli* (*E. coli*) (Yuan et al., 2022; Li C. et al., 2025; Gu et al., 2024). These compositional alterations are not merely passive epiphenomena; rather, they are translated into functional signals that remodel the hepatic immune microenvironment. At the cellular regulatory level, *P. mirabilis* levels are inversely correlated with hepatic Kupffer cell (KC) levels; its increased abundance reduces KC numbers and promotes the migration of CT26 colorectal cancer cells. In contrast, *B. vulgatus* levels are positively correlated with KC levels, and its reduced abundance is associated with suppressed KC proliferation. The two bacteria exert opposing dose-and time-dependent effects on KC proliferation (Yuan et al., 2022). Meanwhile, tumor-resident *E. coli* enhances local lactate production; lactate induces lactylation of retinoic acid-inducible gene 1, suppresses NF-κB signaling, mediates M2 macrophage polarization, and impairs the immunosuppressive activity of regulatory T cells (Tregs) and the antitumor activity of CD8^+^ T cells (Gu et al., 2024). In addition, tumor-secreted cathepsin K (CTSK) binds Toll-like receptor 4 on tumor-associated macrophages (TAMs), stimulates M2 polarization via an mTOR-dependent pathway, and forms a CTSK-mediated positive feedback loop between CRC cells and TAMs (Li et al., 2019). Collectively, these processes indicate that microbiota dysbiosis can drive hepatic pre-metastatic niche formation through multiple immunometabolic signals.

Metabolic reprogramming is another core component of the upstream driver role of microbiota dysbiosis. Gut microbiota dysbiosis profoundly alters the bile acid metabolic profile, including increased production of lithocholic acid (LCA). As a microbiota-derived secondary bile acid, LCA activates TGR5 on hepatic cancer-associated fibroblasts (CAFs) within metastatic lesions, upregulates CCL3 secretion, and subsequently recruits CCR1^+^ myeloid-derived suppressor cells (MDSCs) to the liver via the CCL3-CCR1 axis, thereby establishing an immunosuppressive microenvironment that promotes metastatic colonization and disease progression (Li C. et al., 2025). Targeted metabolomics of serum bile acids further showed that non-absorbable antibiotic treatment upregulated primary bile acids, downregulated secondary bile acids, and increased the CA/βMCA ratio. *In vitro* and *in vivo* experiments confirmed that deoxycholic acid (DCA) accelerates CRC cell proliferation and metastasis (Deng et al., 2022). Therefore, microbial metabolites such as LCA and DCA serve as key mediators that translate compositional changes in the microbiota into pro-metastatic functional signals.

Barrier disruption and local inflammation further amplify the aforementioned upstream driver effects. Bertocchi et al. (2021) found that tumor-resident *E. coli* disrupts the gut vascular barrier (GVB) through the bacterial virulence regulator VirF, allowing bacteria to disseminate to the liver via the portal circulation and promoting immune dysregulation, innate immune cell accumulation, and a pro-tumor inflammatory milieu; PV-1, a marker of GVB impairment, is associated with hepatic bacterial dissemination and metachronous distant metastasis. Sakamoto et al. (2021) showed that Fn-positive colorectal cancer liver metastasis (CRLM) tissues exhibit significantly lower CD8^+^ T-cell density and significantly higher MDSC density, suggesting that Fn is associated with an immunosuppressive microenvironment. Yu et al. (2025) further found that Fn infection downregulates miR-5692a, increases IL-8 expression, activates the ERK pathway, and induces epithelial-mesenchymal transition (EMT), thereby promoting CRC liver metastasis. In summary, microbiota dysbiosis and its associated metabolic reprogramming constitute upstream drivers of hepatic pre-metastatic niche formation by regulating KCs, MDSCs, M2 macrophages, neutrophils, neutrophil extracellular traps (NETs), and EMT, thereby promoting CRLM progression.

In summary, gut microbiota dysbiosis and metabolic reprogramming collectively transform the gut-liver axis into a production line that continuously outputs pathological signals, characterized by elevated levels of pro-PMN metabolites such as LPS, secondary bile acids, and trimethylamine N-oxide, alongside reduced levels of protective metabolites such as butyrate and acetate. However, these upstream signals do not directly constitute the functional features of the PMN; rather, they influence PMN formation by acting on distinct hepatic cell populations.

## 3 Signal perception and cellular reprogramming: complex cellular regulatory networks orchestrate formation of hepatic PMN

Whether metastatic tumor cells can survive and proliferate in the liver depends on early hepatic conditioning by the primary tumor, PMN cell interactions, and a permissive microenvironment. Intestinal barrier dysfunction enables microbiota-derived products and metabolites to enter the liver via the portal vein, forming the PMN signal layer. Various liver cells act as sensors; these signals reprogram them from a homeostatic to a pro-metastatic state, forming the effector layer. Hepatic PMN cell subpopulations include hepatocytes, hepatic interstitial cells, and immune cells (Brodt, 2016) (Supplementary Table 1).

### 3.1 Liver parenchymal cells-hepatocytes

Hepatocytes reside between the hepatic capillaries and blood sinusoids within the hepatic lobules. As the main cellular components and functional units of the liver, they constitute 60% of hepatic cells and 80% of the total volume, executing essential functions in bilirubin metabolism, bile secretion, protein synthesis (e.g., coagulation factors and plasma proteins), and metabolic homeostasis. This anatomical positioning makes hepatocytes the first parenchymal cell type exposed to gut-derived factors via the portal vein. In the hepatic PMN, hepatocytes actively sense primary tumor and gut microbial signals, remodeling the liver microenvironment before tumor cell arrival (Chen et al., 2020).

Wang Z. et al. (2023) demonstrated that fatty liver upregulates hepatocyte Rab27a, promoting secretion of EVs enriched with miR-25-3p, miR-92a-3p, and miR-103-3p. These EVs suppress LATS2 in CRC cells, activating YAP-CYR61 signaling, which promotes M2-like macrophage infiltration and CD8^+^ T-cell exhaustion, establishing a pre-metastatic liver microenvironment (Wang Z. et al., 2023). Primary tumor-derived factors can directly induce EMT in hepatocytes. Pucci et al. (2023) demonstrated that CRC-derived small extracellular vesicles induce TGFβ1-mediated EMT in hepatocytes, thereby generating a pro-fibrotic and pro-tumorigenic hepatic milieu conducive to metastatic seeding. Hepatocyte metabolic integrity serves as a critical barrier against metastasis. Fan et al. reported that hepatocyte PHB1 and MATα1 preserve mitochondrial function and methylation homeostasis; hepatocyte-specific Phb1 or Mat1a knockout markedly increases hepatic metastasis, indicating that loss of hepatocyte metabolic defense permits PMN formation (Fan et al., 2024).

Gut microbiota dysbiosis generates an altered pool of gut-derived metabolites-including secondary bile acids, tryptophan derivatives, short-chain fatty acids, and LPS-that drain through the portal vein and directly act on hepatocytes. In parallel, loss of bacterial bile salt hydrolase activity leads to the accumulation of conjugated primary bile acids, such as taurocholic acid, which alters hepatocyte FXR signaling and promotes the recruitment of immunosuppressive myeloid cells (Zheng et al., 2025). These reprogrammed hepatocytes release EVs, secrete IL-6, TNF-α, and CXCL12, and upregulate MMP2/MMP9, collectively establishing an immunosuppressive, matrix-remodeled hepatic PMN (Yu et al., 2022).

### 3.2 Hepatic interstitial cells

#### 3.2.1 Liver sinusoidal endothelial cells (LSECs)

LSECs are highly specialized, discontinuous, interfacing blood and the space of Disse. Comprising approximately 50% of liver non-parenchymal cells, they are characterized by sieve-plate fenestrae (100–200 nm), absence of a continuous basement membrane, and high endocytic/scavenging activity (Jiang et al., 2022). As the first barrier to gut-derived molecules and circulating tumor cells, LSECs regulate vascular permeability, immune trafficking, and tumor cell transmigration in the hepatic PMN.

Under homeostasis, LSECs express low ICAM-1 and minimal VCAM-1/E-selectin. During PMN formation, tumor-derived factors and inflammation upregulate these adhesion molecules, promoting tumor cell arrest and transmigration. ICAM-1 blockade or silencing reduced liver tumor retention by 50% and metastatic burden by 75%, identifying LSEC ICAM-1 as a key mediator of pro-metastatic stromal responses (Benedicto et al., 2019). Cancer-derived extracellular vesicles carrying AMIGO2 further enhance tumor–endothelial adhesion, priming LSECs for metastatic capture (Izutsu et al., 2022). Metabolic liver disease and inflammation induce LSEC defenestration, capillarization, and increased permeability. Steatohepatitis-associated vascular remodeling promotes melanoma liver metastasis, indicating that pre-existing LSEC dysfunction creates a pro-metastatic milieu (Hoffmann et al., 2025). Cancer cells also induce intercellular gaps in LSECs via proinflammatory paracrine signals, facilitating tumor transmigration (Huu Hoang et al., 2022). Lyve-1 deficiency in LSECs/liver lymphatic endothelium alters the hepatic immune microenvironment and melanoma liver metastasis susceptibility (Jauch et al., 2022). LSECs regulate hepatic immunity via chemokine presentation. LSEC-derived CXCL16 recruits antitumor CXCR6^+^ natural killer T (NKT) cells; its loss impairs NKT surveillance. LSECs also constitutively express PD-L1 and cross-present antigens to CD8^+^ T cells without co-stimulation, inducing T-cell hyporesponsiveness (Ma et al., 2018).

Dysbiosis-driven shifts in the primary-to-secondary bile acid ratio reprogram LSEC chemokine secretion and immune surveillance. Gut barrier disruption increases portal LPS, which acts via TLR4 on both tumor cells and LSECs. LPS-stimulated TLR4/MD2-expressing CRC cells activate PI3K/AKT and β1 integrin–mediated adhesion to hepatic sinusoidal endothelium, doubling tumor capture and increasing liver metastasis (Hsu et al., 2011).

#### 3.2.2 Kupffer cells (KCs)

Kupffer cells are the liver’s resident macrophages, constituting 20–35% of liver non-parenchymal cells and the largest tissue-resident macrophage population. Positioned along the sinusoidal lumen, they adhere to endothelium and are directly exposed to portal and arterial blood. Functionally, KCs are the liver’s primary phagocytic sentinels.

In the hepatic PMN, KCs sense tumor-derived and gut microbial signals, polarize toward pro-tumorigenic phenotypes, and secrete cytokines that remodel the hepatic microenvironment. Tumor-derived EV uptake and TGF-β-driven fibrosis. PDAC-derived exosomes enriched in MIF are preferentially taken up by hepatic KCs, triggering TGF-β secretion. TGF-β activates hepatic stellate cells (HSCs) to produce fibronectin, recruiting bone marrow-derived macrophages and neutrophils and initiating a fibrotic, immunosuppressive PMN (Costa-Silva et al., 2015). KCs are reprogrammed toward an M2-like phenotype during PMN formation. Liu et al. demonstrated that highly metastatic CRC cells secrete CXCL16, which binds CXCR6 on KCs and activates PI3K/AKT1/FOXO3a, transcriptionally upregulating CD206 and driving M2 polarization. M2-like KCs further secrete CXCL16 in a positive feedback loop; inhibiting this axis attenuates CRC liver metastasis (Liu et al., 2025). Tang et al. confirmed that high-metastatic CRC cells induce KC M2 polarization, and bufalin reverses this via the SRC-3/IL-6 pathway. M2-polarized KCs secrete IL-10 and TGF-β, suppressing CD8^+^ T-cell cytotoxicity. (Tang et al., 2024).

LPS is sensed by KCs via TLR4/MD2, activating MyD88/TRIF-NF-κB signaling and inducing TNF-α, IL-6, and chemotactic factors. This chronic activation upregulates endothelial adhesion molecules, enhances myeloid recruitment, and suppresses antitumor immunity. Yuan et al. showed that gut microbiota alteration remodels the liver immune microenvironment, including KC polarization: *B. vulgatus* enrichment increased KC proliferation and reduced metastasis, whereas *P. mirabilis* expansion decreased KC numbers and created a permissive environment. Antibiotic treatment altered KC composition and metastasis susceptibility (Yuan et al., 2022).

### 3.3 Immune cells

#### 3.3.1 Natural killer (NK) cells

Natural killer cells constitute 30–50% of hepatic lymphocytes, far exceeding their peripheral blood proportion. In the hepatic PMN, NK cells enforce DTC dormancy through IFN-γ, but their numbers contract and cytotoxicity declines during PMN maturation, creating a permissive immune window (Correia et al., 2021).

Correia et al. (2021) demonstrated that liver-resident NK cells sustain breast cancer DTC dormancy via IFN-γ. Exit from dormancy coincides with NK cell contraction and activated hepatic stellate cell (aHSC) accumulation; aHSC-derived CXCL12 engages CXCR4 on NK cells, driving quiescence. IL-15-based therapy preserving NK cells prevents hepatic metastasis (Correia et al., 2021). Yang et al. demonstrated that CRC-derived EVs downregulate NKG2D on NK cells without altering their numbers, abrogating tumor surveillance. NK cell depletion abolished the metastatic difference between EV-educated and control livers, confirming EV-induced NK dysfunction as requisite for PMN formation (Yang et al., 2023). Liu et al. identified the HLA-E: CD94-NKG2A axis as a checkpoint enabling circulating tumor cell escape from NK killing; tumor HLA-E engages the inhibitory receptor CD94/NKG2A, suppressing cytotoxic granule release (Liu et al., 2023). The hepatic PMN also imposes metabolic constraints on NK cells. Zhou et al. reported that ORM2, upregulated in the pre-metastatic liver, binds ACVR1 on NK cells and activates Hippo signaling, suppressing serine/one-carbon metabolism essential for NK cytotoxicity (Zhou et al., 2026).

Gut dysbiosis-through iso-LCA accumulation and LPS translocation-directly impairs hepatic NK cell cytotoxicity and survival, collapsing the innate immune barrier that enforces DTC dormancy. LPS modulates NK activation via TLR4/MyD88, while short-chain fatty acid depletion further compromises NK metabolic fitness (Wei et al., 2025).

#### 3.3.2 T lymphocytes

T lymphocytes are key effectors of adaptive immunity, capable of killing tumor cells and reducing metastatic potential. The changes in the quantity and function of T cells in tumor tissues can reflect the intensity and overall level of the body’s anti-tumor response to a certain extent (Sasada and Suekane, 2011). In hepatic PMN, different T cell subsets play opposing roles.

Sun et al. (2023) identified CHSY1 as inhibiting SDH, causing succinate accumulation and PI3K/AKT/HIF-1α activation, which upregulates PD-L1 on CRC cells. PD-L1 engages PD-1 on CD8^+^ T cells, inducing exhaustion (reduced IFN-γ, TNF-α, granzyme B). CHSY1 knockdown or artemisinin restores CD8^+^ T-cell cytotoxicity and reduces metastatic burden (Sun et al., 2023). The lactic acid-rich PMN activates Tregs via the MCT1-NFAT1-PD-1 axis. Kumagai et al. demonstrated that Tregs take up lactic acid via MCT1, promoting NFAT1 nuclear translocation and PD-1 upregulation. Liver metastases with high glycolysis exhibit expanded PD-1^+^ Tregs, and MCT1 blockade synergizes with anti-PD-1 therapy (Kumagai et al., 2022). Primary tumor-derived EVs also impair CD8^+^ T cells before tumor arrival. CRC-derived EVs upregulate PD-1 and TIM-3 on CD8^+^ T cells, suppress cytokine secretion, and induce antigen-specific T-cell apoptosis, pre-emptively disabling adaptive immunity (Zhu et al., 2024).

Under dysbiosis, bile acid–metabolizing bacteria expand, shifting the bile acid pool toward immunomodulatory secondary bile acids that skew intrahepatic T cells toward Treg dominance and away from Th17/effector responses (Hang et al., 2019). Conversely, *R. intestinalis*-derived butyrate activates CD8^+^ T cells (granzyme B, IFN-γ) and boosts anti–PD-1 efficacy; butyrate depletion in dysbiosis promotes PD-1 upregulation and exhaustion (Kang et al., 2023).

#### 3.3.3 Myeloid-derived suppressor cells (MDSCs)

Myeloid-derived suppressor cells are pathologically activated immature myeloid cells that expand under chronic inflammation and suppress antitumor immunity. They comprise monocytic MDSCs (Mo-MDSCs) and granulocytic MDSCs (G-MDSCs), also termed polymorphonuclear MDSCs (PMN-MDSCs). During PMN formation, tumor-derived factors drive MDSC mobilization from bone marrow and recruitment to hepatic sinusoids, where they establish a dominant immunosuppressive compartment (Zhao et al., 2020).

Lin et al. established that sphingosine-1-phosphate receptor 1 (S1PR1) upregulation in CRC cells activates signal transducer and activator of transcription 3 (STAT3), which transcriptionally induces IL-6. Elevated IL-6 in turn stimulates systemic MDSC expansion and their recruitment to the liver, where these MDSCs form a pre-metastatic immunosuppressive niche that promotes organ-specific liver metastasis. (Lin et al., 2019). Zeng et al. confirmed that MDSCs accumulate in the pre-metastatic liver of CRC-bearing mice and in stage I-III CRC patients. Metabolic reprogramming-upregulated glycolysis and HIF-1 activation-promotes MDSC migration to the pre-metastatic liver, with PGK1 implicated. Hepatic MDSCs secrete immunosuppressive factors and upregulate immune checkpoints, shaping the PMN (Zeng et al., 2021). Ren et al. demonstrated that Mo-MDSCs secrete CCL7, which activates CCR3 on disseminated CRC cells and promotes exit from dormancy, driving metastatic progression from latency. (Ren et al., 2021). Recruited MDSCs suppress immunity via conserved mechanisms: Mo-MDSCs produce iNOS/NO, while G-MDSCs generate reactive oxygen species (ROS) and Arg-1 (depleting L-arginine). Both secrete TGF-β and IL-10, inhibiting CD8^+^ T and NK cells and promoting Treg expansion (Yin et al., 2020).

Li C. et al. (2025) demonstrated that microbiota-derived secondary bile acids (particularly LCA) activate TGR5 on CAFs, inducing chemokine secretion that recruits MDSCs to the liver. This microbiota–bile acid–TGR5–MDSC axis links gut microbial metabolism directly to hepatic PMN formation (Li C. et al., 2025).

#### 3.3.4 Neutrophils

Neutrophils are among the first leukocytes recruited to inflammatory sites, including tumors, and in liver metastasis, they predominantly promote progression through the release of NETs and angiogenic factors.

Xu et al. (2025) Identified three neutrophil subpopulations in CRC liver metastasis. Tumor-derived GM-CSF converts antitumor neutrophils to a pro-tumor state via AKT-NF-κB-NFAT5; these neutrophils accumulate in hepatic sinusoids and release NETs upon CXCR2 ligand stimulation, preparing the liver for metastasis (Xu et al., 2025). Okamoto et al. (2023) demonstrated that activated neutrophils undergo NETosis, releasing chromatin decorated with neutrophil elastase, MPO, and citrullinated histones. NET-derived elastase activates ERK in CRC cells, promoting proliferation and metastatic colonization. Wang et al. (2021) identified P2RX1-negative neutrophils accumulating in PDAC liver metastases. These cells exhibit elevated Nrf2, metabolic reprogramming toward oxidative phosphorylation, and high PD-L1/Arg-1, potently suppressing CD8^+^ T cells in a PD-L1/PD-1-dependent manner (Wang et al., 2021). Huang et al. (2025) demonstrated that neutrophils drive breast cancer liver metastasis via methionine metabolism mediated by MAT2A, highlighting neutrophil metabolic adaptation.

Kong et al. (2023) demonstrated that Fn-enriched in CRC and liver metastases-triggers NET formation via TLR4-ROS and NOD1/2 signaling in neutrophils. Fn-induced NETs stimulate angiogenesis and metastasis; high Fn tissues show greater neutrophil infiltration and NETs (Kong et al., 2023).

#### 3.3.5 Macrophages

Beyond resident KCs (Section 3.2.2), monocyte-derived macrophages are recruited during hepatic PMN formation. Tumor-derived chemokines drive massive monocyte recruitment to perisinusoidal and periportal regions. Macrophages promote PMN formation through M2 polarization, T cell exhaustion, and stromal remodeling.

Tu et al. (2021) demonstrated that TAM recruitment and M2 polarization are increased in CRC liver metastases versus primary tumors, dependent on the TCF4-CCL2-CCR2 axis. Li S. et al. (2025) demonstrated that colon cancer exosomal HSP90B1 is taken up by hepatic macrophages, polarizing M1 to M2 and initiating liver PMN formation; M2 macrophages secrete pro-tumorigenic factors that prime the liver for seeding. Liang et al. (2024) confirmed that exosomal miR-106a-5p from highly metastatic CRC cells induces macrophage M2 polarization, driving liver metastasis. Trehan et al. (2025) identified SPP1^+^ macrophages in liver metastases that cause tumor-specific T cell exhaustion. These SPP1^+^ macrophages, together with M2 TAMs, secrete IL-10, TGF-β, and Arg-1, inhibiting CD8^+^ T-cell cytotoxicity (Trehan et al., 2025).

Li et al. (2019) demonstrated that gut microbiota stimulation induces the secretion of cathepsin K (CTSK), which mediates TLR4-dependent M2 macrophage polarization and enhances tumor cell invasion and metastatic progression (Figure 1).

**Figure 1:**
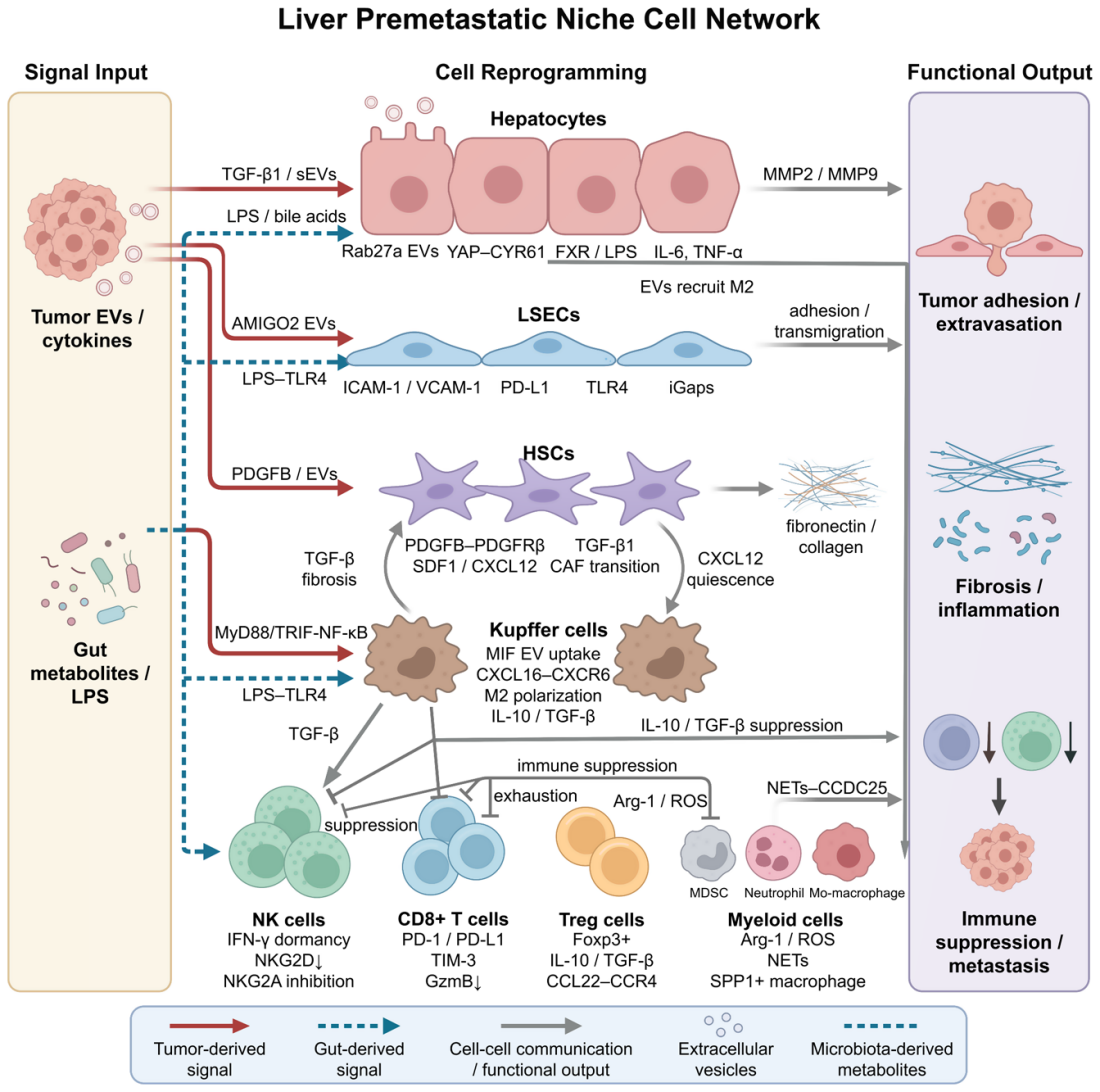
Cellular regulatory network of the hepatic pre‑metastatic niche. Tumor-derived extracellular vesicles/cytokines and gut-derived metabolites/LPS reprogram hepatocytes, LSECs, HSCs, and Kupffer cells. These cells then communicate with NK cells, CD8^+^ T cells, Treg cells, and myeloid cells, driving tumor adhesion/extravasation, fibrosis/inflammation, and immune suppression that facilitate metastatic colonization.

## 4 Microbiota-derived signals drive hepatic PMN formation

The gut–liver axis operates as a directional signal flow: barrier disruption permits microbial entry, distinct metabolite classes then reprogram the hepatic stromal and immune compartments, and these signals converge on a shared recruitment effector layer. The section is organized along this causal chain rather than by isolated pathway (Supplementary Table 2).

### 4.1 Gut barrier disruption and microbial translocation: the entry gate

The hepatic PMN requires gut-derived material to reach the liver. In tumor-bearing hosts, both the epithelial barrier and the GVB are compromised, enabling bacteria and microbial products to disseminate through the portal vein. Tumor-resident *E. coli* disrupt the GVB through the virulence regulator VirF, allowing intestinal bacteria to disseminate to the liver before overt metastatic colonization and to initiate local innate immune activation (Bertocchi et al., 2021). This translocation is the necessary entry point: without it, the metabolite signals discussed below do not reach hepatic parenchymal, stromal, or immune cells. In this model, the vascular permeability marker PV-1 is elevated and correlates with bacterial hepatic dissemination and metachronous distant metastasis (Bertocchi et al., 2021).

### 4.2 Microbial signals prime hepatic inflammation, stromal remodeling, and immune suppression

Once translocated, LPS-a major outer-membrane component of Gram-negative bacteria-engages TLR4 across KCs, hepatocytes, and liver sinusoidal endothelial cells, converging on NF-κB-driven pro-inflammatory transcription. *In vivo*, systemic LPS exposure enhances colorectal carcinoma cell adhesion to the liver within 1.5 h, accompanied by rapid loss of the tight-junction protein ZO-1 and disruption of hepatic microvascular integrity; KC-derived ROS mediates this endothelial damage, as macrophage depletion or ROS scavenging abolishes both the intercellular gaps and the increase in tumor-cell adhesion (Gül et al., 2012). This primes the hepatic endothelium for adhesion, licenses chemokine output, and sets the inflammatory baseline on which downstream metabolites act. Rather than a linear terminal cascade, LPS functions as an inflammatory amplifier: it does not by itself recruit suppressive cells but sensitizes the liver to do so.

The bile acid pool is among the microbially transformed metabolites most closely linked to hepatic PMN formation. In liver-metastatic CRC patients, gut bacteria with bile salt hydrolase (BSH) activity are significantly depleted, and conjugated primary bile acids-notably taurocholic acid (TCA)-accumulate in the circulation (Zheng et al., 2025). Mechanistically, TCA upregulates TIMP1 in CRC cells, which in turn stimulates CXCL5/CXCR2-mediated neutrophil recruitment to the pre-metastatic liver; unconjugated primary and secondary bile acids counteract neutrophil migration induced by TCA *in vitro* and abolish TCA-promoted neutrophil recruitment and liver metastasis *in vivo* (Zheng et al., 2025). Independently, high hepatic TCA uptake activates HSCs through TGR5, expanding stromal fibroblasts and hepatic MDSC, neutrophil, and macrophage proportions (Zheng et al., 2022). Cholestasis-induced phenotypic transformation of hepatic neutrophils has further been reported to contribute to immune escape during CRC liver metastasis (Sun et al., 2024). Collectively, these studies link microbial BSH activity to stromal remodeling and neutrophilic infiltration in the pre-metastatic liver through TCA/TIMP1/CXCL5 and TGR5/HSC signaling.

Beyond diffusible metabolites, pathobiont species that persist in the tumor-bearing gut can reach and remodel the hepatic immune compartment. Fn, enriched in CRC and its liver metastases, exaggerates liver metastasis in tumor-bearing mice and alters the hepatic immune compartment: it expands liver MDSCs and Tregs while reducing NK cells, Th17 cells, and total CD8^+^ T-cell infiltration. Sustained *F. nucleatum* exposure also reduces gut microbial diversity (Yin et al., 2022). In contrast to LPS, which signals through a broadly expressed receptor, this remodeling is species-specific and combines direct immune-cell alteration with secondary changes in commensal metabolism.

### 4.3 Reshaping of the hepatic and intrametastatic microbiota niche

Once translocated, gut-associated bacteria are not merely transient passengers in the liver; they establish an intrametastatic microbial community whose structure correlates with local immune state and metastatic outcome. In a clinical cohort of 44 patients with CRLM and 85 patients without liver metastasis, profiling of intratumoral microbiota identified three reproducible intratumoral microbial community subtypes (IMCSs). IMCS1, associated with sugar metabolism, correlated with T-cell activation and the most favorable disease-free survival (DFS; median 22 months); IMCS2, associated with protein metabolism, correlated with NK-cell activation (median DFS 12 months); IMCS3, associated with lipid metabolism and enriched for *Fusobacterium*, *Faecalibacterium*, *Methylobacterium*, and *Agrobacterium*, was pauci-immune, showed the highest Ki-67/p53 expression, and had the poorest prognosis (median DFS 10 months) (Yan et al., 2025). The progressive loss of T/NK activation across IMCS1 → IMCS3 indicates that the intrametastatic microbiota composition itself stratifies whether the hepatic microenvironment remains immune-competent or becomes immunosuppressive.

This intrametastatic community is not passive. Tumor-resident *E. coli* within CRLM enhances local lactate production, induces RIG-I lactylation, suppresses NF-κB/NLRP3 signaling, and polarizes hepatic macrophages toward an M2-like phenotype, directly remodeling the local immune compartment (Gu et al., 2024). Together with earlier evidence that bacteria disseminate to the liver before overt metastatic colonization and recruit myeloid cells with extracellular matrix deposition (Section 4.1), these findings support a model in which the liver is colonized by a structured, tumor-adapted microbial community that actively shapes the immune and stromal properties of the pre-metastatic and early metastatic niche, rather than being merely exposed to scattered bacterial products.

## 5 Diagnosis and treatment strategies for liver pre-metastasis

### 5.1 Diagnosis

The liver lacks pain nerves and retains substantial functional reserve, so early hepatic metastases are clinically silent, and conventional surveillance often detects lesions only after they become radiologically evident. Conventional tumor-marker testing and cross-sectional imaging remain the standard surveillance tools, but circulating markers have limited specificity and CT/PET-CT poorly detect small or hypometabolic lesions; these modalities identify established metastases rather than the PMN itself. Multimodal strategies that combine refined imaging, liquid biopsy, and circulating immune or serological markers are therefore being explored to detect the hepatic PMN before overt metastasis.

Liquid biopsy offers a non-invasive, earlier window. Exosomal content correlates with TNM staging and enables earlier detection than imaging (Li S. et al., 2023). Exosomal liquid biopsy has emerged as a promising non-invasive approach. Circulating exosomal miRNAs reported in colorectal cancer include serum exosomal miR-122 (Sun et al., 2020), exosomal miRNA panels (Yan et al., 2017), and exosomal miR-6803-5p (Yan et al., 2018).

Circulating immune and serological markers extend this panel and, in several cases, link directly to PMN biology. Elevated blood NETs, which are themselves mediators of hepatic PMN formation, predict liver-metastasis risk in early breast cancer (Yang et al., 2020). In CRC, peripheral monocyte count independently predicts liver metastasis (Yang et al., 2025). Serologically, lipopolysaccharide-binding protein (LBP), which signals through TLR4/NF-κB to promote a hepatic fibrotic niche, is a candidate marker for gastric cancer liver metastasis (Xie et al., 2023). In CRC, a random-forest model combining lactate dehydrogenase (LDH), carbohydrate antigen 19–9 (CA19-9), alanine aminotransferase (ALT), carcinoembryonic antigen (CEA), total bilirubin (TBIL), and the albumin-to-globulin ratio (AGR) predicted liver metastasis in a cohort of 865 patients (Feng et al., 2025).

Collectively, these findings highlight the need for integrated diagnostics combining imaging refinement, exosomal microRNA (miRNA) profiling, and immune-clinical marker analysis. It is necessary to validate these biomarkers in prospective, multi-center cohorts to establish their sensitivity and specificity for early PMN detection before overt metastasis (Table 1).

**Table 1:** Emerging diagnostic strategies for the hepatic pre-metastatic niche.

**Modality/biomarker**	**Sample**	**Cancer type**	**Study design**	**Sample size**	**What is detected**	**Principal finding**	**Relation to PMN biology**	**Causal grade**	**Evidence model**	**Ref**
Exosomal content; circulating exosomal miRNAs (miR-122, miRNA panel, miR-6803-5p)	Serum/plasma EVs	CRC	NR	NR	Correlation with TNM stage; earlier detection than imaging	Exosomal content correlates with TNM staging and enables earlier detection than imaging. Circulating exosomal miRNAs reported in CRC (serum miR-122, miRNA panel, miR-6803-5p) have undefined expression-change directions	Candidate liquid-biopsy correlate; not shown to detect the hepatic PMN	A	HO	Li S. et al. (2023), Sun et al. (2020), Yan et al. (2017), and Yan et al. (2018)
Circulating NETs (NET-DNA)	Peripheral blood	Early breast cancer	Clinical cohort + mechanistic work	NR	Predicts future liver metastasis	Elevated circulating NETs predict liver-metastasis risk; NET-DNA binds CCDC25 and activates ILK-β-parvin signaling	Direct PMN mediator (analyte is itself a niche driver)	A (biomarker); D (mechanism)	HO + AM	Yang et al. (2020)
Peripheral monocyte count	Peripheral blood	CRC	Retrospective cohort	n = 7,187	Predicts future liver metastasis	Peripheral monocyte count independently associated with liver-metastasis risk	PMN-associated correlate (plausible given CCR2-dependent monocyte recruitment)	A	HO	Yang et al. (2025)
Serum LBP	Peripheral blood	Gastric cancer	Clinical cohort + mouse model	NR	Predicts/promotes liver metastasis	Gastric cancer-derived LBP drives intrahepatic fibrotic PMN formation via TLR4/NF-κB	Direct PMN mediator linking diagnosis to the gut-liver axis	A (biomarker); D (mechanism)	HO + AM	Xie et al. (2023)
LDH, CA19-9, ALT, CEA, TBIL and AGR (random-forest model)	Peripheral blood	CRC	Retrospective machine-learning cohort	n = 865	Predicts future liver metastasis	Combined model predicted liver metastasis	PMN-associated correlate reflecting hepatic functional/inflammatory state	A	HO	Feng et al. (2025)

See Supplementary Table 1 for complete definitions of the two-axis grading scheme, codes and PMN-hallmark abbreviations.

### 5.2 Pharmacological and pathway-targeted interventions

Most interventions discussed in this section are supported by preclinical evidence in murine models; none is approved specifically for hepatic PMN prevention.

Chemotherapy can itself condition the liver for metastasis. Administration of oxaliplatin before the onset of liver metastasis accelerates tumor colonization in murine models by shifting the hepatic immune microenvironment toward immunosuppression, with reduced macrophage and functional CD8^+^ T-cell numbers and the appearance of immunosuppressive macrophages. This effect is reversed by T-cell supplementation, indicating that hepatic immune conditioning is a determinant of chemotherapy-associated metastatic risk and that timing of adjuvant treatment warrants attention (Ma et al., 2023).

Pathway-directed inhibitors remain preclinical. The SGK1 inhibitor GSK-650394 reduces IL-6/STAT3- and ERK-mediated NET formation and PMN-MDSC infiltration, and inhibits liver metastasis in ischemia–reperfusion models (Li X. et al., 2023). Galunisertib, a TGF-β receptor I kinase inhibitor, blocks TGF-β/Smad signaling and attenuates hepatic fibrosis and PMN formation in gastric cancer liver-metastasis models (Xie et al., 2023). The FXR agonist obeticholic acid reduces hepatic inflammation and reverses the immunosuppressive liver microenvironment in CRC models (Li Z. et al., 2025). A galactose-targeted lipid-calcium-phosphate nanocarrier delivering miR-122 regulates metastasis-associated genes, increases the CD8^+^/CD4^+^ T-cell ratio, and reduces immunosuppressive cell infiltration (Sendi et al., 2022). These agents have not been tested prospectively for PMN prevention in patients, but are highly anticipated.

### 5.3 Microbiota-targeted interventions for the hepatic PMN

Antibiotics: indirect effects that are difficult to evaluate. Oral vancomycin depletes Gram-positive bacteria that mediate primary-to-secondary bile-acid conversion; in murine models this is associated with hepatic NKT-cell accumulation and reduced liver tumor growth (Ma et al., 2018). Oral neomycin reduces intestinal Gram-negative load and portal LPS exposure, which attenuates TLR4-driven CXCL1 production and decreases CXCR2^+^ PMN-MDSC recruitment to the liver (Zhang et al., 2021). These are indirect, taxa-level effects rather than direct PMN targeting. Broad-spectrum antibiotics also cause collateral dysbiosis, select for antimicrobial resistance, alter bile-acid metabolism, and reduce response to immune-checkpoint blockade. Their use as anti-metastatic therapy is therefore not supported by current evidence; any future strategy would require narrow-spectrum agents, defined microbial targets, controlled timing, and reversibility.

Dietary, prebiotic, and postbiotic approaches. Restriction of capsaicin-associated diets restores mucin-associated taxa (*Akkermansia*, Muribaculaceae), alters bile-acid metabolism, and restores hepatic NKT-cell accumulation (Cheng et al., 2023). Inulin and cellulose, alone or as mixtures, expand *Bifidobacterium*, *Lactobacillus*, and Lachnospiraceae, restore acetate, propionate, isobutyrate, and butyrate, and regulate EMT (Wang C. et al., 2023). Sodium butyrate supplementation in CRC liver-metastatic mice restores microbial composition, reduces hepatic Tregs while increasing NKT and Th17 cells, and suppresses liver metastasis (Ma et al., 2020).

Microbiota-modulating natural products. PRM1201 inhibits CRC metastasis by reshaping the gut microbiota and enriching short-chain fatty acids in murine models (Jia et al., 2024). Echinacoside suppresses CRC metastasis by modulating the gut microbiota and inhibiting PI3K/AKT signaling (Wei et al., 2024). Yanggan Yizhong decoction, a traditional herbal formula, prevents CRC liver metastasis by remodeling gut bile-acid metabolism and reducing hepatic MDSC recruitment; the anti-metastatic effect was transferred by fecal microbiota transplantation, establishing a causal role for the microbiota (Xie et al., 2025). These studies are concordant in showing that microbiota remodeling can suppress hepatic metastasis, but the causal taxa and metabolites remain undefined.

No microbiota-directed intervention has been tested specifically for hepatic PMN prevention, but human data in metastatic CRC inform the broader translational context. A phase II, open-label, single-arm trial in refractory microsatellite-stable metastatic CRC combined fecal microbiota transplantation with the anti-PD-1 antibody tislelizumab and the VEGFR inhibitor fruquintinib, reporting a median progression-free survival of 9.6 months and overall survival of 13.7 months, with enrichment of favorable taxa among responders (Zhao et al., 2023). Concurrently, retrospective cohort analyses consistently show that broad-spectrum antibiotic exposure before or during immune-checkpoint blockade reduces progression-free and overall survival across tumor types (hazard ratios for death approximately 1.4–2.0), with the strongest association for fluoroquinolones, β-lactams, and carbapenems (Petrelli et al., 2025). These data underscore that microbiota-based interventions must be evaluated alongside concurrent chemotherapy and immunotherapy. Patient selection based on baseline microbiota composition, optimal timing relative to adjuvant treatment, long-term safety and reversibility, and whether defined postbiotics or engineered consortia outperform undefined dietary, herbal, or antibiotic interventions remain unresolved (Table 2).

**Table 2:** Interventions that modify hepatic pre-metastatic niche formation: pathway inhibitors, microbiota-targeted strategies and treatment-induced conditioning.

**Strategy class**	**Intervention**	**Target**	**Cancer**	**Model**	**Timing vs metastasis**	**Mechanism**	**Endpoint**	**Effect direction**	**Causal grade**	**Evidence model**	**Translational status**	**Ref**
Iatrogenic niche conditioning	Oxaliplatin before metastasis onset	Hepatic immune compartment	CRC	Mouse	Before seeding (PMN window)	Shifts the hepatic immune microenvironment toward immunosuppression (fewer macrophages and functional CD8⁺ T cells; appearance of immunosuppressive macrophages); reversed by T-cell supplementation	Accelerated hepatic colonization	Pro-metastatic	D	AM	Standard-of-care agent; PMN-conditioning effect is preclinical	Ma et al. (2023)
Pathway-directed inhibitor	GSK-650394	SGK1	CRC	Mouse (hepatic ischemia–reperfusion)	Before seeding (PMN window)	Reduces IL-6/STAT3- and ERK-mediated NET formation and PMN-MDSC infiltration	NETs; PMN-MDSC infiltration; liver metastasis	Anti-metastatic	D	AM	Preclinical; not tested for PMN prevention clinically	Li X. et al. (2023)
	Galunisertib	TGF-β receptor I kinase	Gastric cancer	Mouse (GC liver-metastasis)	Before seeding (PMN window)	Blocks TGF-β/Smad signaling and attenuates LBP-driven fibrotic hepatic PMN formation	Hepatic fibrosis; PMN formation; liver metastasis	Anti-metastatic	D	AM	In clinical development for other indications; not tested for PMN prevention	Xie et al. (2023)
	Obeticholic acid	FXR agonist	CRC	Mouse (as summarised)	Before seeding (PMN window)	Reduces hepatic inflammation and reverses the immunosuppressive liver microenvironment	Hepatic inflammation; immunosuppressive microenvironment	Anti-metastatic	I	RV	Approved for primary biliary cholangitis; currently cited source is a review, so graded I pending a primary study	Li Z. et al. (2025)
	Galactose-targeted LCP nanoparticle delivering miR-122	Metastasis-associated gene network	CRC	Mouse	Before/during seeding	Regulates metastasis-associated genes, increases CD8⁺/CD4⁺ T-cell ratio and reduces immunosuppressive infiltration	T-cell ratio; immunosuppressive infiltration; liver metastasis	Anti-metastatic	D	AM	Preclinical	Sendi et al. (2022)
Antibiotic (taxa-level, indirect)	Oral vancomycin	Gram-positive bacteria mediating primary-to-secondary bile-acid conversion	Primary liver cancer	Mouse (gnotobiotic)	Primary tumor stage	Raises primary bile acids, upregulates LSEC CXCL16 and promotes hepatic NKT-cell accumulation	Hepatic NKT number; primary liver tumor growth	Anti-tumor	D (primary liver cancer); I (hepatic PMN)	AG	Not recommended clinically: collateral dysbiosis, resistance, impaired immune-checkpoint response	Ma et al. (2018)
	Oral neomycin	Gram-negative load/portal LPS	Cholangiocarcinoma	Mouse (germ-free/antibiotic)	PT to PMN	Attenuates hepatocyte TLR4-driven CXCL1 and decreases CXCR2⁺ PMN-MDSC recruitment	Hepatic PMN-MDSC recruitment; tumor growth	Anti-tumor	D (cholangiocarcinoma); I (hepatic PMN)	AG	Same clinical caveats as vancomycin	Zhang et al. (2021)
	Non-absorbable antibiotics	Secondary bile-acid-producing bacteria	CRC	Mouse	Before seeding (PMN window)	Raises primary bile acids, lowers secondary bile acids and reduces DCA-driven proliferation/metastasis	Serum bile-acid profile; liver-metastatic burden	Anti-metastatic	D	AG	Preclinical; causal taxa undefined	Deng et al. (2022)
Antibiotic (harm signal)	Broad-spectrum antibiotics around immune-checkpoint blockade	Whole gut community	Multiple tumors	Human retrospective cohorts (review)	Established metastasis	Antibiotic-induced dysbiosis impairs microbiota-dependent checkpoint-blockade efficacy	PFS/OS; HR for death ~1.4–2.0 (strongest for fluoroquinolones, β-lactams, carbapenems)	Harmful	A	HO	Argues against antibiotics as anti-metastatic therapy	Petrelli et al. (2025)
Dietary / prebiotic	Restriction of capsaicin-associated diet	*Akkermansia*, Muribaculaceae	CRC	Mouse	Before seeding (PMN window)	Restores mucin-associated taxa, alters bile-acid metabolism and restores hepatic NKT accumulation	Hepatic NKT accumulation; metastatic burden	Anti-metastatic	D	AG	Preclinical	Cheng et al. (2023)
	Inulin, cellulose, or mixture	Bifidobacterium, Lactobacillus, Lachnospiraceae	CRC	Mouse (prevention)	Prevention	Expands short-chain fatty acid producers and restores acetate, propionate, isobutyrate and butyrate; regulates EMT	Short-chain fatty acids; EMT; liver metastasis	Anti-metastatic	D	AG	Preclinical	Wang C. et al. (2023)
Postbiotic	Sodium butyrate	Short-chain fatty acids/microbial composition	CRC	Mouse (CRC liver metastasis)	PMN / MET	Restores microbial composition, reduces hepatic Tregs and increases NKT and Th17 cells	Hepatic immune composition; liver-metastatic burden	Anti-metastatic	D	AG	Preclinical; defined postbiotic	Ma et al. (2020)
Microbiota-modulating natural product	PRM1201	Gut microbiota; short-chain fatty acids	CRC	Mouse	PT to metastasis	Reshapes gut microbiota and enriches short-chain fatty acids	Microbiota composition; short-chain fatty acids; metastasis	Anti-metastatic	M	AG	Preclinical; causal taxa undefined	Jia et al. (2024)
	Echinacoside	Gut microbiota; PI3K/AKT	CRC	Mouse	PT to metastasis	Modulates gut microbiota and suppresses PI3K/AKT signaling	Microbiota composition; metastasis	Anti-metastatic	M	AG	Preclinical	Wei et al. (2024)
	Yanggan Yizhong decoction	Gut bile-acid metabolism; hepatic MDSCs	CRC	Mouse + FMT transfer	Prevention	Remodels gut bile-acid metabolism and reduces hepatic MDSC recruitment; anti-metastatic effect transferred by FMT	Bile-acid profile; hepatic MDSCs; liver metastasis; FMT transferability	Anti-metastatic	D	AG	Preclinical; FMT transfer establishes microbial causality	Xie et al. (2025)
Microbiota transplantation (human)	FMT + tislelizumab + fruquintinib (RENMIN-215)	Gut microbiota; PD-1; VEGFR	MSS metastatic CRC	Human (phase II, single-arm)	Established metastasis (not PMN window)	FMT combined with anti-PD-1 and VEGFR inhibition; responders enriched for favorable taxa	Median PFS 9.6 months; median OS 13.7 months	Anti-tumor	D (efficacy signal)	HI	Only human interventional entry; open-label, no concurrent control; addresses established metastasis	Zhao et al. (2023)
Host immune-preserving	IL-15-based therapy	Liver-resident NK cells	Breast cancer	Mouse (hepatic dormancy)	PMN / DOR	Preserves the NK compartment that enforces IFN-γ-dependent DTC dormancy	NK maintenance; prevention of overt hepatic metastasis	Anti-metastatic	D	AM	Preclinical; targets the dormancy window	Correia et al. (2021)
	Bufalin	SRC-3/IL-6; KC M2 polarization	CRC	Mouse + in vitro	SEED / MET	Reverses KC M2-type polarization induced by highly metastatic colon cancer cells	KC phenotype; CD8⁺ cytotoxicity; liver metastasis	Anti-metastatic	M	AM + IV	Preclinical	Tang et al. (2024)
	Artemisinin (or CHSY1 knockdown)	CHSY1-succinate-HIF-1α-PD-L1	CRC	Mouse	SEED / MET	Restores CD8⁺ T-cell cytotoxicity by relieving succinate-driven PD-L1 upregulation	Cytokine/granzyme output; metastatic burden	Anti-metastatic	D	AM	Preclinical	Sun et al. (2023)
	MCT1 blockade + anti-PD-1	Treg MCT1-NFAT1-PD-1 axis	Multiple, including liver metastases	Mouse + human tissue	MET	Blocks lactate uptake by Tregs, limiting NFAT1-driven PD-1 upregulation	PD-1⁺ Treg frequency; synergy with anti-PD-1	Anti-metastatic	D	AM + HT	Preclinical	Kumagai et al. (2022)
	GM-CSF or CXCR2 blockade	Pro-tumor neutrophils/NETs	CRC	Mouse + human tissue	Before seeding (PMN window)	Prevents GM-CSF-driven neutrophil conversion and CXCR2-triggered NET release in hepatic sinusoids	Neutrophil subsets; NETs; metastatic burden	Anti-metastatic	D	AM + HT	Preclinical	Xu et al. (2025)

See Supplementary Table 1 for complete definitions of the two-axis grading scheme, codes and PMN-hallmark abbreviations.

## 6 Conclusion

Liver metastasis constitutes a major fatal factor for malignant tumor patients, and the evidence synthesized here supports the view that the gut-liver axis is not a passive conduit but an active architect of the hepatic PMN. Primary tumor-associated dysbiosis alters the luminal metabolome; barrier disruption allows microbial products and selected taxa to reach the liver; and hepatocytes, liver sinusoidal endothelial cells, KCs, and recruited myeloid and lymphoid populations sense these signals and reprogram toward a pro-metastatic phenotype. Bile acids, secondary bile acid derivatives, short-chain fatty acids, lipopolysaccharide, and tumor-resident bacteria converge on overlapping inflammatory, immunosuppressive, and matrix-remodeling pathways, producing the cardinal PMN features described throughout this review.

Most mechanistic evidence derives from colorectal cancer, and the dominant taxa-*F. nucleatum*, tumor-resident *E. coli*, *Proteus mirabilis*, and *Bacteroides vulgatus*-are specific to CRC-associated dysbiosis. Features such as gut vascular barrier disruption, bile-salt hydrolase deficiency, and secondary bile acid accumulation appear broadly relevant, whereas the specific microbial signatures, dominant metabolites, and cellular effectors in gastric, pancreatic, breast, and lung cancer are less well defined. Cross-tumor extrapolation should be cautious until equivalent multi-omic and gnotobiotic evidence is available for other primary sites.

In addition, the hepatic PMN is not only a staging ground for immediate outgrowth; it may also be the soil in which DTCs persist for years before reactivating. In the liver, NK cells sustain DTC dormancy through IFN-γ signaling, and exit from dormancy coincides with NK-cell contraction and the accumulation of activated hepatic stellate cells that secrete CXCL12 and drive NK quiescence (Correia et al., 2021). Neutrophil extracellular traps further reawaken dormant DTCs by cleaving laminin and exposing integrin-activating epitopes, and NRF2-dependent metabolic reprogramming supports reactivated cells. The clinical corollary is direct: persistent or re-established hepatic inflammation, fibrosis, and immunosuppression after primary tumor treatment may keep the liver permissive for delayed relapse, even after adjuvant therapy has eradicated the bulk disease. This raises the possibility that microbiota-targeted interception should be considered not only at the stage of radiologically detectable liver metastases but also during the dormancy window, when DTCs are clinically occult but the hepatic microenvironment remains conditioned.

Translational progress nonetheless remains constrained. Diagnostic biomarkers-exosomal miRNAs, circulating NETs, LBP, and machine-learning combinations-derive largely from retrospective cohorts and have not been validated for PMN detection prospectively. Therapeutically, pathway inhibitors, antibiotics, dietary fibers, postbiotics, and microbiota-modulating natural products are supported almost entirely by murine models; broad-spectrum antibiotics carry documented risks of dysbiosis, resistance, and impaired immune-checkpoint inhibitor response, and no microbiota-directed agent has been tested specifically for hepatic PMN prevention. Future work should use gnotobiotic models with defined consortia to establish causal taxa and metabolites, stratify findings by primary tumor type, and test interception strategies prospectively in patients at high risk of delayed liver recurrence.

## Glossary

AGR: Albumin-to-globulin ratio
aHSC: Activated hepatic stellate cell
ALT: Alanine aminotransferase
BSH: Bile salt hydrolase
CA19-9: Carbohydrate antigen 19–9
CAF: Cancer-associated fibroblast
CEA: Carcinoembryonic antigen
CRC: Colorectal cancer
CRLM: Colorectal cancer liver metastasis
CT: Computed tomography
DCA: Deoxycholic acid
DFS: Disease-free survival
DTC: Disseminated tumor cell
EMT: Epithelial-mesenchymal transition
EV: Extracellular vesicle
*E. coli*: *Escherichia coli*
Fn: *Fusobacterium nucleatum*
G-MDSC: Granulocytic myeloid-derived suppressor cell
GVB: Gut vascular barrier
HSC: Hepatic stellate cell
3-IAA: Indole-3-acetic acid
IMCS: Intratumoral microbial community subtype
KC: Kupffer cell
LBP: Lipopolysaccharide-binding protein
LCA: Lithocholic acid
LDH: Lactate dehydrogenase
LPS: Lipopolysaccharide
LSEC: Liver sinusoidal endothelial cell
MDSC: Myeloid-derived suppressor cell
miRNA: MicroRNA
Mo-MDSC: Monocytic myeloid-derived suppressor cell
NET: Neutrophil extracellular trap
NK: Natural killer
NKT: Natural killer T
PDAC: Pancreatic ductal adenocarcinoma
PET: Positron emission tomography
PMN: Pre-metastatic niche
PMN-MDSC: Polymorphonuclear myeloid-derived suppressor cell
ROS: Reactive oxygen species
TAM: Tumor-associated macrophage
TBIL: Total bilirubin
TCA: Taurocholic acid
Th17: T helper 17 cell
TNM: Tumor-node-metastasis
Treg: Regulatory T cell.
