# Supplementary material for: Gut-liver axis in liver pre-metastatic niche formation: immune networks, microbial, and metabolic and emerging therapeutic opportunities
Source: Front Microbiol. 2026 Oct 15;17:1931054. doi: 10.3389/fmicb.2026.1931054 (PMC13717628; doi:10.3389/fmicb.2026.1931054)
Supplement: Supplementary file 1 [file Table_1.docx]

**Supplementary** **Table 1** Cellular components of the hepatic pre-metastatic niche: mechanisms, experimental basis and strength of causal evidence

| **Hepatic cell population** | **Primary tumor type** | **Species** | **Experimental model** | **Stage window** | **Intervention / perturbation** | **Mechanism** | **Endpoint measured** | **PMN hallmark** | **Causal grade** | **Evidence model** | **Ref** |
| --- | --- | --- | --- | --- | --- | --- | --- | --- | --- | --- | --- |
| Hepatocytes | CRC | Mouse | Fatty-liver host + CRC metastasis model | PMN | Modulation of hepatocyte EV output | Fatty liver upregulates hepatocyte Rab27a and secretion of EVs enriched in miR-25-3p, miR-92a-3p and miR-103-3p; these EVs suppress LATS2 and activate YAP-CYR61 signaling, promoting M2-like macrophage infiltration and CD8⁺ T-cell exhaustion | Pre-metastatic liver microenvironment; macrophage and CD8⁺ T-cell phenotypes | IMS, MTB | D | AM | [33] |
|  | CRC | Human cells | Hepatocytes exposed to CRC-derived small EVs | PMN | sEV exposure (in vitro) | CRC-derived small EVs induce TGF-β1-mediated EMT of hepatocytes, generating a pro-fibrotic, pro-tumorigenic milieu | EMT and fibrogenic phenotype (no in vivo metastasis endpoint) | ECM | M | IV | [34] |
|  | CRC / primary liver cancer | Mouse | Hepatocyte-specific Phb1 or Mat1a knockout | PMN | Cell-type-specific genetic knockout | Hepatocyte PHB1 and MATα1 preserve mitochondrial function and methylation homeostasis; loss of this metabolic defence permits hepatic metastasis | Hepatic metastatic burden increased in knockout livers | MTB | D | AM | [35] |
|  | CRC | Human + Mouse | Patient bile-acid metabolomics + murine bile-acid supplementation/rescue | PMN | BSH-active bacteria depleted; TCA supplementation; unconjugated bile-acid rescue | Loss of bacterial BSH activity leads to accumulation of conjugated primary bile acids such as TCA, which alters hepatocyte FXR signaling and promotes recruitment of immunosuppressive myeloid cells; TCA upregulates tumor TIMP1 and CXCL5/CXCR2-mediated neutrophil recruitment | Circulating bile-acid profile; hepatic neutrophil recruitment; liver-metastatic burden (abolished by unconjugated bile-acid rescue) | IMS, INF | D | HO + AG | [36] |
| LSECs | CRC | Mouse | Liver-metastasis model with ICAM-1 blockade/silencing | SEED | ICAM-1 antibody blockade or silencing | tumor-derived factors and inflammation upregulate LSEC ICAM-1, promoting tumor-cell arrest and transmigration | Liver tumor retention reduced by ~50%; metastatic burden reduced by ~75% | VAS, INF | D | AM | [39] |
|  | Gastric cancer / CRC | Mouse + cells | Cancer-cell-derived EV pre-conditioning | SEED | AMIGO2-containing EVs | Cancer-derived EVs carrying AMIGO2 enhance tumor-endothelial adhesion, priming LSECs for metastatic capture | tumor-endothelial adhesion | VAS | M | IV + AM | [40] |
|  | Melanoma | Mouse | Steatohepatitis followed by melanoma liver colonization | NTL | Dietary induction of steatohepatitis | Steatohepatitis-associated LSEC defenestration, capillarisation and increased permeability create a pro-metastatic vascular niche | Melanoma liver-metastatic burden | VAS | I | AM | [41] |
|  | CRC | Mouse + cells | tumor-cell/LSEC co-culture and in vivo transmigration | SEED | Proinflammatory paracrine signaling | Cancer cells induce intercellular gaps in LSECs via proinflammatory paracrine signals, facilitating tumor transmigration | Sinusoidal gap formation; tumor transmigration | VAS | M | AM + IV | [42] |
|  | Melanoma | Mouse | Lyve1 knockout | NTL / SEED | Lyve1 gene knockout | Lyve-1 deficiency in LSECs/liver lymphatic endothelium alters the hepatic immune microenvironment and melanoma liver-metastasis susceptibility | Hepatic immune composition; metastasis susceptibility | IMS | M | AM | [43] |
|  | Primary liver cancer | Mouse | Gnotobiotic / antibiotic bile-acid manipulation | PT / NTL | Vancomycin depletion of Gram-positive bacteria | Primary bile acids regulate LSEC CXCL16 expression, controlling chemokine-dependent accumulation of hepatic CXCR6⁺ NKT cells | Hepatic NKT-cell accumulation; primary liver tumor growth (liver metastasis not assessed) | IMS | I | AG | [44] |
|  | CRC | Human cells + Mouse | LPS challenge with TLR4 pathway inhibition | SEED | LPS; TLR4/MD2 pathway inhibition | Portal LPS engages TLR4/MD2 on CRC cells, activating PI3K/AKT and β1-integrin-mediated adhesion to hepatic sinusoidal endothelium | Hepatic tumor-cell capture approximately doubled; increased liver metastasis | VAS, INF | D | IV + AM | [45] |
| HSCs / hepatic CAFs | PDAC | Mouse | Exosome education + PDAC model | PMN | MIF knockdown in tumor-derived exosomes | PDAC-derived exosomes enriched in MIF are taken up by KCs, triggering TGF-β secretion; TGF-β activates HSCs to produce fibronectin and recruit bone marrow-derived macrophages and neutrophils | Hepatic fibronectin deposition; myeloid recruitment; metastatic burden | ECM, IMS | D | AM | [46] |
|  | Breast cancer | Mouse | Hepatic DTC dormancy model | DOR | NK-cell depletion; IL-15-based therapy; aHSC modulation | aHSCs accumulate at dormancy exit and secrete CXCL12, which engages CXCR4 on liver-resident NK cells and drives NK quiescence, releasing DTCs from IFN-γ-enforced dormancy | NK-cell number/function; dormancy exit; overt hepatic metastasis (prevented by IL-15 therapy) | DRM, IMS | D | AM | [49] |
|  | CRC | Mouse | Conjugated bile-acid loading | PMN | TCA administration | High hepatic TCA uptake activates HSCs through TGR5, expanding stromal fibroblasts and hepatic MDSC, neutrophil and macrophage proportions | Stromal fibroblast expansion; hepatic myeloid proportions | ECM, IMS | M | AM | [74] |
|  | CRC | Mouse | LCA supplementation with CCL3/CCR1 blockade | PMN / MET | LCA supplementation; chemokine-axis blockade | Microbiota-derived LCA activates TGR5 on hepatic CAFs, upregulates CCL3 and recruits CCR1⁺ MDSCs via the CCL3-CCR1 axis | Hepatic MDSC infiltration; metastatic colonization and burden | IMS | D | AG | [24] |
| KCs (liver-resident macrophages) | CRC | Mouse | Highly metastatic CRC liver-colonization model | PMN / SEED | CXCL16/CXCR6 axis inhibition | Highly metastatic CRC cells secrete CXCL16, which binds CXCR6 on KCs and activates PI3K/AKT1/FOXO3a, upregulating CD206 and driving M2-like polarization; M2-like KCs further secrete CXCL16 in a positive-feedback loop | KC polarization; CRC liver-metastatic burden (attenuated by axis inhibition) | IMS | D | AM + IV | [47] |
|  | CRC | Mouse | Colon cancer liver-metastasis model | SEED / MET | Bufalin | Highly metastatic colon cancer cells induce KC M2-type polarization via SRC-3/IL-6; M2-polarized KCs secrete IL-10 and TGF-β and suppress CD8⁺ T-cell cytotoxicity; bufalin reverses this polarization | KC phenotype; CD8⁺ cytotoxicity; liver metastasis | IMS | M | AM + IV | [48] |
|  | Colon carcinoma | Rat | Systemic LPS with macrophage depletion / ROS scavenging | SEED | Macrophage depletion; ROS scavenging | LPS acts on KC TLR4; KC-derived ROS causes ZO-1 loss and disrupts hepatic microvascular integrity, opening intercellular gaps | tumor-cell adhesion to liver within 1.5 h; sinusoidal gaps (abolished by macrophage depletion or ROS scavenging) | INF, VAS | D | AM | [73] |
|  | CRC | Mouse | FMT and antibiotic treatment; CT26 model | PMN | FMT; antibiotic depletion | *Proteus mirabilis* abundance correlates inversely and *Bacteroides vulgatus* positively with hepatic KC number; the two exert opposing dose- and time-dependent effects on KC proliferation | KC number/proliferation; metastasis susceptibility | IMS | M | AG | [23] |
| Monocyte-derived macrophages / TAMs | CRC | Mouse + human tissue | Liver-metastasis model with TCF4 knockdown | MET | TCF4 knockdown | TCF4 drives CCL2 production and CCR2-dependent monocyte recruitment; TAM density and M2-like polarization are higher in liver metastases than in matched primary tumors | TAM density; M2 markers; hepatic metastatic burden | IMS | D | AM + HT | [69] |
|  | CRC | Mouse | Exosome education of the pre-metastatic liver | PMN | HSP90B1 knockdown in tumor exosomes | Colon cancer exosomal HSP90B1 is taken up by hepatic macrophages and polarizes M1 toward M2, initiating PMN formation (resident KC versus recruited monocyte-derived macrophage not distinguished) | Macrophage polarization; PMN formation; metastatic seeding | IMS | D | AM | [70] |
|  | CRC | Mouse | Exosomal miRNA transfer | SEED | miR-106a-5p inhibition | Exosomal miR-106a-5p from highly metastatic CRC cells induces macrophage M2 polarization (ontogeny not resolved) | Macrophage polarization; liver-metastatic burden | IMS | D | AM | [71] |
|  | CRC and other primaries | Human tissue | Single-cell profiling of resected liver metastases | MET | None (descriptive) | SPP1⁺ macrophages, together with M2-like TAMs, secrete IL-10, TGF-β and Arg-1 and are associated with exhaustion of tumor-specific T cells | Macrophage and T-cell transcriptional states | IMS | A | HT | [72] |
|  | CRC | Mouse + human | Microbiota manipulation with CTSK loss of function | PT / MET | Antibiotic depletion; CTSK inhibition/knockout | Gut microbiota stimulation induces tumor secretion of CTSK, which binds TLR4 on TAMs and drives M2 polarization via an mTOR-dependent pathway | M2 polarization; invasion; metastatic progression | IMS | D | AG + AM | [26] |
|  | CRC | Mouse + human CRLM | Intratumoral bacteria; antibiotic depletion | MET | Antibiotic depletion of tumor-resident bacteria | tumor-resident *E. coli* within CRLM enhances local lactate, induces RIG-I lactylation, suppresses NF-κB/NLRP3 signaling and polarizes hepatic macrophages toward M2-like (intrametastatic compartment) | Macrophage phenotype; Treg and CD8⁺ function; metastatic burden | IMS | M | AG + HT | [25] |
| NK cells | Breast cancer | Mouse | Hepatic DTC dormancy model | DOR | NK-cell depletion; IL-15-based therapy | Liver-resident NK cells sustain DTC dormancy via IFN-γ; dormancy exit coincides with NK contraction driven by aHSC-derived CXCL12-CXCR4 signaling | Dormancy maintenance versus exit; overt hepatic metastasis prevented by IL-15 therapy | DRM | D | AM | [49] |
|  | CRC | Mouse | EV education of the liver + NK depletion | PMN | CRC-derived EVs; NK-cell depletion | CRC-derived EVs downregulate NKG2D on NK cells without altering NK numbers, abrogating tumor surveillance; NK depletion abolishes the metastatic difference between EV-educated and control livers | NKG2D expression; metastatic burden | IMS | D | AM | [50] |
|  | CRC and other primaries | Mouse + human | CTC/NK assays; NKG2A blockade | SEED | NKG2A blockade | tumor HLA-E engages CD94/NKG2A on NK cells and suppresses cytotoxic granule release, enabling CTC escape from NK surveillance | NK cytotoxicity; CTC survival; metastasis | IMS | D | AM + IV | [51] |
|  | CRC | Mouse | ORM2 gain- and loss-of-function | PMN | ORM2 knockdown / overexpression | ORM2 upregulated in the pre-metastatic liver binds ACVR1 on NK cells and activates Hippo signaling, suppressing serine/one-carbon metabolism required for NK cytotoxicity | NK cytotoxicity; hepatic metastatic burden | MTB, IMS | D | AM | [52] |
|  | Primary liver cancer | Mouse | AKR1D1-dependent bile-acid metabolism | PT / NTL | AKR1D1 manipulation | Bile-acid metabolism governs hepatic NK cytotoxicity; by extension, dysbiosis-driven iso-LCA accumulation, LPS translocation and short-chain fatty acid depletion are proposed to impair hepatic NK fitness | NK cytotoxicity; primary liver tumor progression (liver metastasis not assessed) | IMS | I | AM | [9] |
| T lymphocytes | CRC | Mouse | CHSY1 knockdown; artemisinin | SEED / MET | CHSY1 knockdown; artemisinin | CHSY1 inhibits SDH, causing succinate accumulation and PI3K/AKT/HIF-1α activation that upregulates tumor PD-L1; PD-L1 engages PD-1 on CD8⁺ T cells and induces exhaustion | IFN-γ, TNF-α, granzyme B; metastatic burden reduced by CHSY1 knockdown or artemisinin | IMS | D | AM + IV | [54] |
|  | Multiple, including liver metastases | Mouse + human | High-glycolysis models; MCT1 blockade + anti-PD-1 | MET | MCT1 blockade; anti-PD-1 | Tregs take up lactic acid via MCT1, promoting NFAT1 nuclear translocation and PD-1 upregulation; high-glycolysis liver metastases show expanded PD-1⁺ Treg populations | PD-1⁺ Treg frequency; synergy with anti-PD-1 | IMS, MTB | D | AM + HT | [55] |
|  | Not tumor-related | Mouse | Gnotobiotic mice fed bile-acid derivatives | NTL | Metabolite supplementation | Secondary bile-acid derivatives control Th17 and Treg differentiation; extrapolated here to intrahepatic T-cell skewing under dysbiosis | Intestinal Th17/Treg balance (no tumor or liver-metastasis endpoint) | IMS | I | AG | [57] |
|  | CRC | Mouse | Mono-colonization with *Roseburia intestinalis* + butyrate | PT / MET | Mono-colonization; butyrate supplementation | *R. intestinalis*-derived butyrate activates CD8⁺ T cells (granzyme B, IFN-γ) and boosts anti-PD-1 efficacy; butyrate depletion promotes PD-1 upregulation and exhaustion | CD8⁺ effector function; tumor burden; anti-PD-1 efficacy (hepatic PMN not specifically assessed) | IMS | D (CD8⁺ activation and anti-PD-1); I (hepatic PMN) | AG | [58] |
| MDSCs (Mo-MDSC / PMN-MDSC) | CRC | Mouse | S1PR1 gain- and loss-of-function in CRC cells | PMN | S1PR1 overexpression / knockdown | S1PR1 upregulation activates STAT3 and induces IL-6; IL-6 drives systemic MDSC expansion and hepatic recruitment, forming a pre-metastatic immunosuppressive niche | Hepatic MDSC accumulation; organ-specific liver metastasis | IMS | D | AM | [60] |
|  | CRC | Mouse + human | Pre-metastatic liver immune landscape; stage I-III CRC patients | PMN | None (descriptive) | MDSCs accumulate in the pre-metastatic liver; upregulated glycolysis and HIF-1 activation (PGK1 implicated) promote MDSC migration; hepatic MDSCs secrete immunosuppressive factors and upregulate immune checkpoints | Hepatic MDSC frequency; checkpoint expression | IMS, MTB | A | AM + HO | [61] |
|  | CRC | Mouse | Dormancy-to-outgrowth model | DOR | CCL7 neutralization | Mo-MDSCs secrete CCL7, which activates CCR3 on disseminated CRC cells and promotes exit from dormancy | Metastatic progression from latency (prevented by CCL7 inhibition) | DRM | D | AM | [62] |
|  | CRC | Mouse | LCA supplementation with CCL3/CCR1 blockade | PMN / MET | LCA supplementation; chemokine-axis blockade | Microbiota-derived LCA activates TGR5 on hepatic CAFs and induces CCL3, recruiting CCR1⁺ MDSCs | Hepatic MDSC infiltration; metastatic burden | IMS | D | AG | [24] |
|  | Cholangiocarcinoma | Mouse | Germ-free / neomycin-treated mice | PT / PMN | Germ-free rederivation; antibiotic depletion | Gut bacteria and LPS reaching the liver drive hepatocyte TLR4-dependent CXCL1 production and recruit CXCR2⁺ PMN-MDSCs | Hepatic PMN-MDSC accumulation; tumor growth (primary cholangiocarcinoma, not liver metastasis) | IMS | D (cholangiocarcinoma); I (hepatic PMN) | AG | [90] |
| Neutrophils | CRC | Mouse + human tissue | CRC liver-metastasis model; GM-CSF / CXCR2 blockade | PMN | GM-CSF and CXCR2 blockade | tumor-derived GM-CSF converts antitumor neutrophils to a pro-tumor state via AKT-NF-κB-NFAT5; these cells accumulate in hepatic sinusoids and release NETs upon CXCR2-ligand stimulation | Neutrophil subsets; NET formation; metastatic burden | INF, IMS | D | AM + HT | [64] |
|  | CRC | Mouse + cells | NETosis with elastase inhibition | SEED | Neutrophil elastase inhibition | Activated neutrophils undergo NETosis; NET-derived elastase activates ERK in CRC cells, promoting proliferation and metastatic colonization | CRC proliferation; metastatic colonization | INF | M | AM + IV | [65] |
|  | PDAC | Mouse + human | PDAC liver-metastasis model; P2RX1 loss of function | MET | P2rx1 knockout | P2RX1-negative neutrophils accumulate in PDAC liver metastases, show elevated Nrf2, oxidative-phosphorylation reprogramming and high PD-L1/Arg-1, and suppress CD8⁺ T cells in a PD-L1/PD-1-dependent manner | CD8⁺ suppression; metastatic burden | IMS, MTB | D | AM + HT | [66] |
|  | Breast cancer | Mouse | Liver-specific metastatic progression; MAT2A manipulation | SEED / MET | MAT2A inhibition | tumor-associated neutrophils drive liver-specific metastatic progression through methionine metabolism mediated by MAT2A | Liver-specific metastatic progression | MTB | M | AM | [67] |
|  | CRC | Mouse + human tissue | Fn colonization; neutrophil assays | PT / MET | Fn colonization | Fn triggers NET formation via TLR4-ROS and NOD1/2 signaling; Fn-induced NETs stimulate angiogenesis and metastasis; high-Fn tissues show greater neutrophil infiltration and NETs | NET formation; angiogenesis; metastatic burden | INF | D | AG + HT | [68] |
|  | CRC | Human + Mouse | Bile-acid metabolomics + TCA supplementation/rescue | PMN | TCA supplementation; unconjugated bile-acid rescue | TCA upregulates tumor TIMP1, stimulating CXCL5/CXCR2-mediated neutrophil recruitment to the pre-metastatic liver | Hepatic neutrophil recruitment and liver metastasis (abolished by unconjugated bile-acid rescue) | INF, IMS | D | AG + HO | [36] |
|  | CRC | Mouse | Cholestasis model | PMN / MET | Induction of cholestasis | Cholestasis-induced phenotypic transformation of hepatic neutrophils contributes to immune escape during CRC liver metastasis | Hepatic neutrophil phenotype; immune escape; metastasis | IMS | M | AM | [75] |

**General note.** Two‑axis evidence grading is applied across Tables 1‑4.

Axis 1 (evidence model, categorical, no ranking): HI, human interventional trial; HO, human observational cohort; HT, human ex‑vivo tissue; AM, conventional‑microbiota animal model; AG, microbiota‑manipulated animal model; IV, in‑vitro assay; RV, review‑level narrative inference. Axis 2 (causal grade, ranked D > M > A > I): D, direct causal evidence for hepatic pre‑metastatic niche (PMN) formation; M, mechanistic/supportive (incomplete causal chain for hepatic PMN); A, associative/correlative only (no experimental perturbation); I, inferred/extrapolated from non‑PMN experimental settings, not direct PMN evidence. Stage‑window codes: PT, primary tumor present, liver unconditioned; PMN, pre‑metastatic conditioned liver; SEED, disseminated tumor‑cell seeding; MET, established macroscopic liver metastasis; DOR, dormancy of disseminated tumor cells; NTL, non‑tumor‑bearing liver (evidence graded I). PMN hallmark abbreviations: INF, inflammation; IMS, immunosuppression; VAS, vascular remodeling and increased sinusoidal permeability; ECM, extracellular-matrix remodeling and fibrosis; MTB, metabolic reprogramming; DRM, dormancy-outgrowth control.
