# Supplementary material for: Gut-liver axis in liver pre-metastatic niche formation: immune networks, microbial, and metabolic and emerging therapeutic opportunities
Source: Front Microbiol. 2026 Oct 15;17:1931054. doi: 10.3389/fmicb.2026.1931054 (PMC13717628; doi:10.3389/fmicb.2026.1931054)
Supplement: Supplementary file 2 [file Table_2.docx]

**Supplementary Table 2** Gut microbiota and microbial metabolites in hepatic pre-metastatic niche formation: compartment of detection, evidence of translocation, and type of microbiome evidence

| **Microbe or metabolite** | **Primary tumor / disease** | **Microbiome evidence type** | **Compartment detected** | **Translocation to liver demonstrated?** | **Species / system** | **Stage** | **Hepatic target** | **Mechanism / principal finding** | **Endpoint** | **Causal grade** | **Key caveats** | **Ref** |
| --- | --- | --- | --- | --- | --- | --- | --- | --- | --- | --- | --- | --- |
| Fn (species) | CRC | Observational: fecal shotgun metagenomics + metabolomics | Faeces | Not addressed | Human, n=616 colonoscopy cohort (HO) | PT | None (upstream) | Fn continuously elevated from intramucosal carcinoma through advanced stages; branched-chain amino acids/phenylalanine and deoxycholate rise early | Stage-stratified abundance; fecal metabolites | A | No strain typing; no liver-metastasis endpoint | [10] |
| *Atopobium parvulum*, *Actinomyces odontolyticus* | CRC | Observational: fecal shotgun metagenomics | Faeces | Not applicable | Human, same cohort (HO) | PT | None | Co-occurring taxa increased specifically in adenomatous and early cancerous lesions | Stage-stratified abundance | A | Early-lesion markers only | [10] |
| Choline-degradation pathways (up); butyrate producers (down) | CRC | Observational: cross-cohort metagenomic meta-analysis | Faeces | Not applicable | Human, multiple CRC cohorts (HO) | PT | None | Reproducible cross-cohort diagnostic signature | Classifier performance | A | Pathways predicted from gene content | [11] |
| Fn, *Porphyromonas asaccharolytica* (up); *Roseburia*, *Eubacterium* (down) | CRC | Observational: meta-analysis of 8 cohorts | Faeces | Not applicable | Human, 8 cohorts (HO) | PT | None | Globally reproducible CRC-specific fecal signature | Differential abundance | A | Genus/species level; cohort heterogeneity | [12] |
| *Pyramidobacter*, Christensenellaceae_R-7_group, *Romboutsia* (metastatic) vs *Butyricicoccus*, *Ruminococcus_1*, *Coprococcus_2* (non-metastatic) | CRC +/- distant metastasis | Observational: 16S rRNA + untargeted LC-MS metabolomics | Faeces | Not addressed | Human, n=16 vs 16 (HO) | PT / MET | None | Dysbiotic and metabolomic profiles differ between metastatic and non-metastatic CRC | Genus-level abundance; 91 differential metabolites | A | Small sample; 16S genus resolution; metastatic site not restricted to liver | [13] |
| *Limosilactobacillus fermentum*, *Blautia* (down); *Prevotella copri* (up) | Breast cancer (I-III) | Observational: fecal shotgun metagenomics + machine learning | Faeces | Not applicable | Human, n=38 vs 36 (HO) | PT | None | Depletion of beneficial taxa and short-chain fatty acid / purine pathways | AUC 0.78 discovery / 0.73 validation | A | Metabolites largely predicted | [14] |
| Reduced diversity; Clostridiales (up) | Breast cancer | Observational: fecal microbiome | Faeces | Not applicable | Human case-control (HO) | PT | None | Reduced diversity and increased Clostridiales versus controls | Alpha diversity; taxon abundance | A | Limited taxonomic resolution | [15] |
| Stage-dependent taxa (*Lachnospira*, *Blautia*, *Bacteroides*, *Prevotella*, *Ruminococcus gnavus*) | Non-small-cell lung cancer | Observational: 16S rRNA + GC/LC-MS metabolomics | Faeces | Not applicable | Human, n=52 vs 29 (HO) | PT | None | Compositional shifts by TNM stage; quinic acid, phthalic acid and L-lactic acid rise in stage III-IV | Stage-stratified taxa and metabolites | A | Small per-stage subgroups; 16S genus level | [16] |
| *Fusobacteria*, *Bacteroides caccae*, *Streptococcus anginosus* (up); *Bifidobacterium longum*, *Lachnospiraceae* (down) | Gastric cancer | Observational: whole-metagenome shotgun sequencing | Faeces | Not applicable | Human, stage-stratified (HO) | PT | None | Stage-specific microbiome signatures with disease progression | Stage-specific abundance | A | No liver-metastasis endpoint | [17] |
| *Akkermansia*, *Gammaproteobacteria*, *Veillonella* (up) | Gastric cancer | Observational: gut metagenomics | Faeces | Not applicable | Human cohort (HO) | PT | None | Enriched taxa proposed as non-invasive biomarkers | Differential abundance | A | Not externally validated | [18] |
| Multi-taxon fecal signature | PDAC | Observational: multi-center metagenomics | Faeces | Not applicable | Human multi-center (HO) | PT | None | fecal microbiota signature with high specificity for pancreatic cancer | Diagnostic specificity | A | Diagnostic, not metastasis-specific | [19] |
| 3-IAA (microbial tryptophan metabolite) | PDAC | Human observational + germ-free / FMT / metabolite supplementation | Faeces; serum | Not applicable (metabolite) | Human + germ-free/FMT mice (HO + AG) | PT | Neutrophils | High 3-IAA associated with improved chemotherapy response; 3-IAA enhances efficacy via neutrophil myeloperoxidase-dependent ROS metabolism | Chemotherapy response; tumor response with 3-IAA supplementation | D (chemotherapy response); I (hepatic PMN) | Endpoint is chemotherapy efficacy, not hepatic PMN formation | [20] |
| IMCS1-3 (IMCS3 enriched for *Fusobacterium*, *fecalibacterium*, *Methylobacterium*, *Agrobacterium*) | CRLM | Observational: intratumoral microbiota profiling | Metastatic lesion (intratumoral) | Not addressed (route of arrival not tested) | Human, n=44 CRLM vs 85 without LM (HT + HO) | MET | T cells; NK cells | Three IMCS subtypes stratify immune state and prognosis (median DFS 22 / 12 / 10 months) | DFS by IMCS subtype | A | Low-biomass tissue microbiome; contamination controls NR | [77] |
| Fn in metastatic tissue | CRLM | Observational: tissue detection | Primary tumor; metastatic lesion | Not addressed | Human CRLM cohort (HT) | MET | CD8⁺ T cells; MDSCs | Fn-positive CRLM tissues show lower CD8⁺ T-cell density and higher MDSC density | Immune-cell density | A | Association only; live vs dead bacteria not distinguished | [29] |
| *Proteus mirabilis* (up); *Bacteroides vulgatus* (down) | CRC | FMT + antibiotic depletion | Faeces only | Not addressed | Mouse, CT26 (AG) | PMN | KCs | Opposing correlations with hepatic KC number and opposing effects on KC proliferation | KC number; metastasis susceptibility | M | Whole-community FMT; bacteria not shown to reach the liver | [23] |
| tumor-resident *E. coli* (VirF⁺) | CRC | Mono-association; antibiotic depletion; GVB reporter | Primary tumor; portal blood; liver tissue | Yes - live bacteria recovered from liver; PV-1 marks GVB breach | Mouse + human tissue (AG + HT) | PT to PMN | Innate immune cells; endothelium | VirF disrupts the GVB, allowing intestinal bacteria to disseminate to the liver via the portal circulation before overt metastatic colonization and to initiate innate immune activation | Hepatic bacterial load; myeloid accumulation; PV-1 associated with metachronous distant metastasis | D | Strongest experimental translocation evidence in this review | [28] |
| tumor-resident *E. coli* (intrametastatic) | CRLM | Intratumoral detection + antibiotic depletion | Metastatic lesion only | Not addressed (route from gut not shown) | Mouse + human CRLM (AG + HT) | MET | Macrophages; Treg; CD8⁺ T cells | Intrametastatic *E. coli* raises local lactate, induces RIG-I lactylation, suppresses NF-κB/NLRP3 and polarizes macrophages M2-like | Macrophage phenotype; T-cell function; metastatic burden | M | Intrametastatic compartment, distinct from luminal gut bacteria | [25] |
| Antibiotic-sensitive gut community (taxon undefined) | CRC | Antibiotic depletion + CTSK loss of function | Faeces; primary tumor | Not addressed | Mouse + human (AG + AM) | PT / MET | TAMs | Gut microbiota stimulation induces tumor CTSK secretion, driving TLR4-dependent M2 TAM polarization | M2 polarization; invasion; metastasis | D (CTSK); M (microbial identity) | Causal taxon not identified | [26] |
| LCA | CRC | Metabolite supplementation + receptor/chemokine blockade | Portal/hepatic circulation (metabolite) | Not applicable (metabolite) | Mouse (AG) | PMN / MET | Hepatic CAFs to MDSCs | LCA activates TGR5 on hepatic CAFs, upregulates CCL3 and recruits CCR1⁺ MDSCs | Hepatic MDSC infiltration; metastatic burden | D | Producing taxa not defined | [24] |
| DCA / secondary bile-acid pool | CRC | Non-absorbable antibiotics + bile-acid metabolomics + DCA supplementation | Serum bile acids | Not applicable (metabolite) | Mouse + in vitro (AG + IV) | PT / MET | tumor cells | Antibiotics raise primary and lower secondary bile acids; DCA accelerates CRC proliferation and metastasis | Serum bile-acid profile; liver-metastatic burden | D | Causal taxa undefined | [27] |
| BSH-active bacteria (down) leading to TCA accumulation | CRC | Human cohort + murine bile-acid supplementation/rescue | Faeces; circulating/portal bile acids | Inferred (metabolite reaches liver) | Human CRLM + mouse (HO + AG) | PMN | Hepatocytes to neutrophils | BSH-active bacteria depleted in liver-metastatic CRC; TCA accumulates and drives TIMP1-CXCL5/CXCR2 neutrophil recruitment to the pre-metastatic liver | Hepatic neutrophil recruitment and liver metastasis (abolished by unconjugated bile-acid rescue) | D | Human association + murine intervention + metabolite rescue | [36] |
| Conjugated bile acids (high hepatic TCA uptake) | CRC | Metabolite loading | Hepatic bile-acid pool | Not applicable (metabolite) | Mouse (AM) | PMN | HSCs / CAFs | High hepatic TCA uptake activates HSCs via TGR5 and expands stromal and myeloid compartments | Stromal fibroblast and myeloid proportions | M | No metastasis-endpoint rescue; microbiota not manipulated | [74] |
| Fn (single strain) | CRC | Oral gavage of tumor-bearing mice | Faeces; primary tumor; liver metastases | Inferred (detected in metastases; transit not directly shown) | Mouse + human (AG + HT) | PT to MET | MDSCs, Treg, NK, Th17, CD8⁺ T cells | Fn exaggerates liver metastasis and remodeled the hepatic immune compartment (MDSCs/Tregs up; NK/Th17/CD8⁺ down); sustained exposure reduces gut diversity | Hepatic immune profiling; metastatic burden | D | Laboratory strain; strain-level functional divergence possible | [76] |
| Fn | CRC | colonization + neutrophil assays | Primary tumor; liver metastases | Inferred | Mouse + human tissue (AG + HT) | PT / MET | Neutrophils | Fn triggers NET formation via TLR4-ROS and NOD1/2; NETs stimulate angiogenesis and metastasis | NET formation; angiogenesis; metastatic burden | D | Strain not genotyped | [68] |
| Fn | CRC | Infection in vitro and in vivo | Primary tumor | Not addressed | Mouse + cell lines (AM + IV) | PT | tumor cells (not hepatic cells) | Fn downregulates miR-5692a, increases IL-8, activates ERK and induces EMT, promoting CRC liver metastasis | EMT markers; liver metastasis | M | Acts on the primary tumor rather than hepatic PMN cells | [30] |
| LPS | Colon carcinoma | Purified PAMP + macrophage depletion / ROS scavenging | Systemic / portal LPS | Not applicable (bacterial product) | Rat (AM) | SEED | KCs; sinusoidal endothelium | LPS enhances tumor-cell adhesion to liver within 1.5 h with ZO-1 loss; KC-derived ROS mediates endothelial damage | Adhesion and sinusoidal gaps (abolished by KC depletion or ROS scavenging) | D | Acute high-dose exogenous LPS | [73] |
| LPS | CRC | Purified PAMP + TLR4 pathway inhibition | Portal LPS | Not applicable | Human cells + mouse (IV + AM) | SEED | tumor cells; LSECs | LPS-stimulated TLR4/MD2-expressing CRC cells activate PI3K/AKT and β1-integrin-mediated adhesion to hepatic sinusoidal endothelium | Hepatic tumor capture doubled; increased liver metastasis | D | Exogenous LPS rather than endogenous endotoxaemia | [45] |
| Gram-positive bacteria (primary-to-secondary bile-acid conversion) | Primary liver cancer | Vancomycin depletion | Faeces; bile-acid pool | Not applicable (metabolite-mediated) | Mouse (AG) | PT | LSECs to CXCR6⁺ NKT cells | Depleting Gram-positive bacteria raises primary bile acids, upregulates LSEC CXCL16 and increases antitumor NKT accumulation | Hepatic NKT number; primary liver tumor growth | D (primary liver cancer); I (hepatic PMN) | Model is primary liver cancer, not liver metastasis | [44] |
| Gram-negative bacteria / LPS | Cholangiocarcinoma | Germ-free rederivation + neomycin | Gut; portal LPS; liver tissue | Yes - gut-derived bacteria and LPS accumulate in the liver | Mouse (AG) | PT / PMN | Hepatocytes to CXCR2⁺ PMN-MDSCs | Barrier dysfunction allows gut bacteria and LPS to accumulate in the liver, driving hepatocyte TLR4-dependent CXCL1 and CXCR2⁺ PMN-MDSC recruitment | Hepatic PMN-MDSC accumulation; tumor growth | D (cholangiocarcinoma); I (hepatic PMN) | Primary hepatobiliary malignancy rather than liver metastasis | [90] |
| Bile-acid derivatives (3-oxoLCA, isoalloLCA) | Not tumor-related | Metabolite supplementation in gnotobiotic mice | Intestinal lumen | Not applicable | Mouse (AG) | NTL | Th17; Treg | Bile-acid metabolites control Th17 and Treg differentiation; cited as basis for proposed intrahepatic Treg skewing under dysbiosis | Intestinal Th17/Treg balance | I | No tumor, liver or metastasis endpoint | [57] |
| *Roseburia intestinalis* / butyrate | CRC | Mono-colonization + butyrate supplementation | Faeces; gut lumen | Not applicable (metabolite) | Mouse (AG) | PT / MET | CD8⁺ T cells | *R. intestinalis*-derived butyrate activates cytotoxic CD8⁺ T cells and boosts anti-PD-1 efficacy | CD8⁺ effector function; tumor burden; anti-PD-1 efficacy | D (CD8⁺ activation and anti-PD-1); I (hepatic PMN) | Hepatic compartment not specifically interrogated | [58] |
| High-fat-diet-associated dysbiosis (community-level) | CRC | Dietary intervention | Faeces | Not addressed | Mouse (AM) | PMN | Hepatocytes | Reprogrammed hepatocytes release EVs, secrete IL-6, TNF-α, and CXCL12, and upregulate MMP2/MMP9, establishing an immunosuppressive, matrix-remodeled hepatic PMN | Immunosuppressive, matrix-remodeled hepatic PMN | M | Liver mRNA only; hepatocyte EVs not shown | [37] |

Note: See Table 1 for complete definitions of the two-axis grading scheme, codes and PMN-hallmark abbreviations.
